# Supplementary figures and images for: Biological variation in the sizes, shapes and locations of visual cortical areas in the mouse
Source: PLoS One. 2019 May 1;14(5):e0213924. doi: 10.1371/journal.pone.0213924 (PMC6493719; doi:10.1371/journal.pone.0213924)

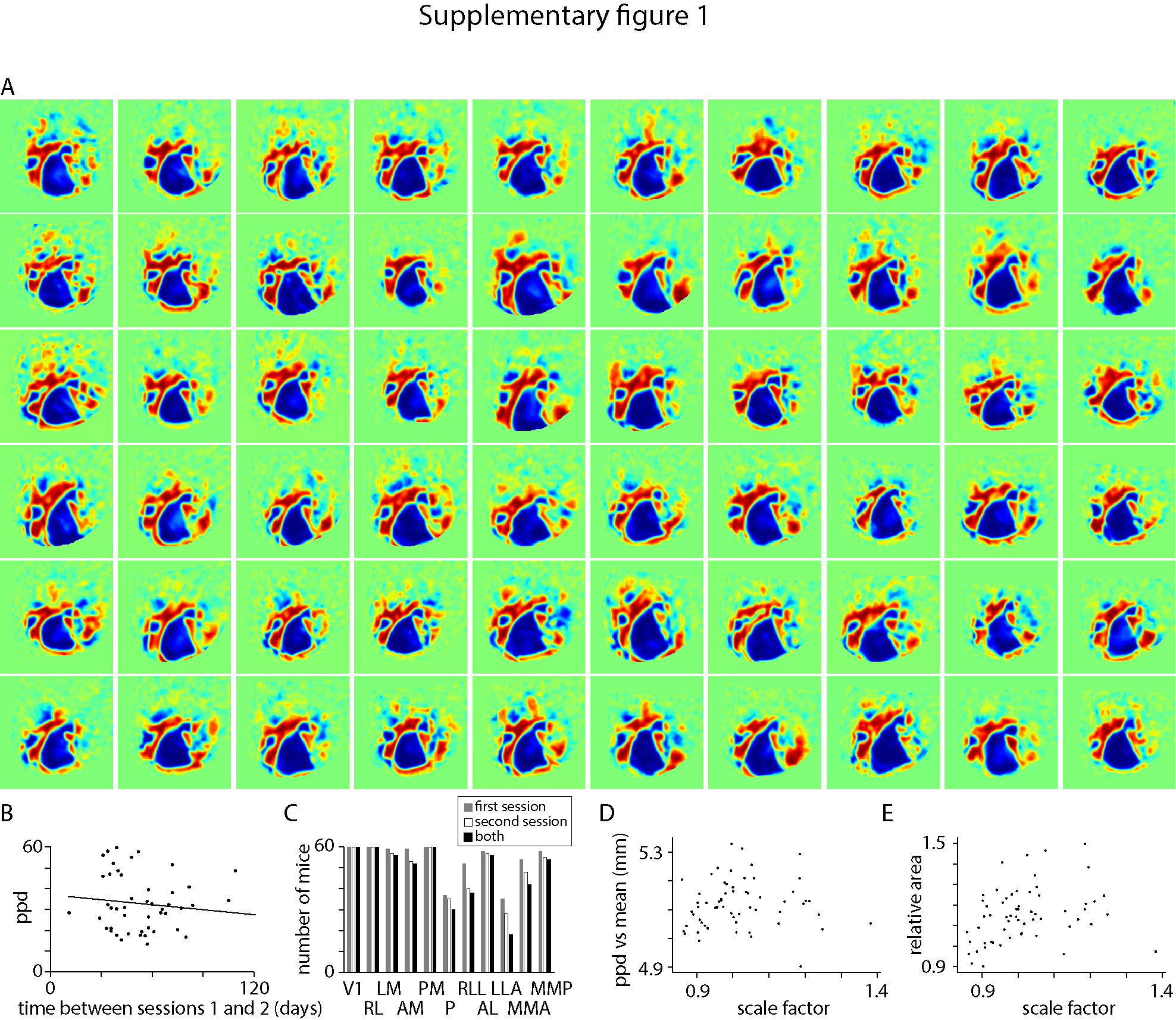

Supplement: S1 Fig — (A) Field sign maps from the first imaging session for each of 60 mice, to illustrate the mouse-to-mouse variability in field sign maps. (B) Plot of paired patch distance (ppd) as a function of time between first and second imaging sessions. Line: best linear fit, slope -0.08. (C) Histogram of the number of times each patch appeared in retinotopic maps. Each patch could occur in a maximum of 60 maps (one for each of 60 mice) for each of the first and second imaging sessions. The right column indicates the number of mice in which the patch was visible in both imaging sessions. (D) Relationship between the scale factor applied to each map and its shape. Shape was measured as the ppd of the map in pairwise comparison with the mean sign map (from 60 mice). Each point represents one map from session 1. (E) Relationship between the scale factor applied to each map and its relative V1 area, where relative V1 area is V1 area in the map divided by the area of V1 in the mean sign map. Each point represents one map from session 1. (TIF) [file pone.0213924.s001.tif]

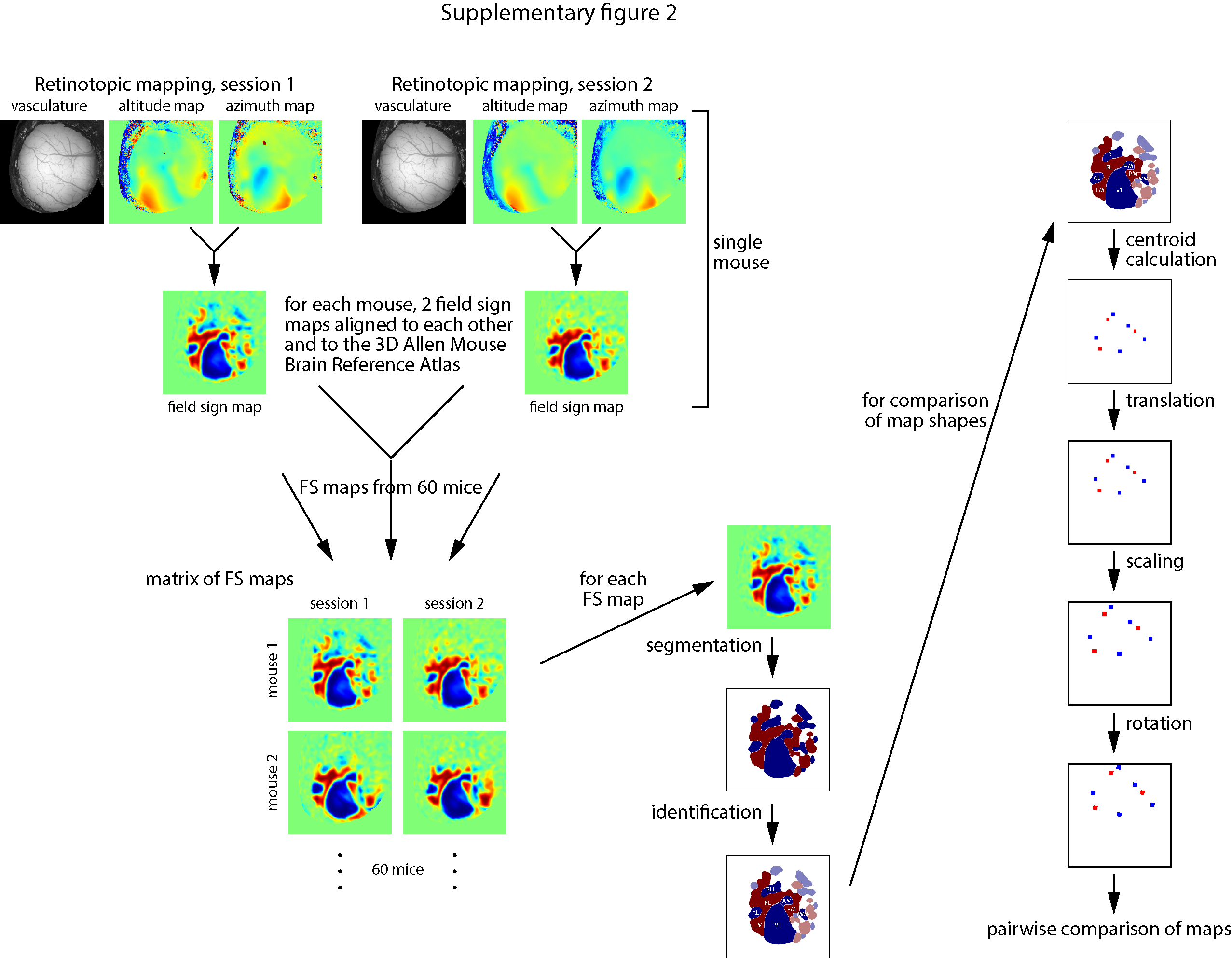

Supplement: S2 Fig — Schematic illustration of the sequence of steps in the core of the analysis. (TIF) [file pone.0213924.s002.tif]

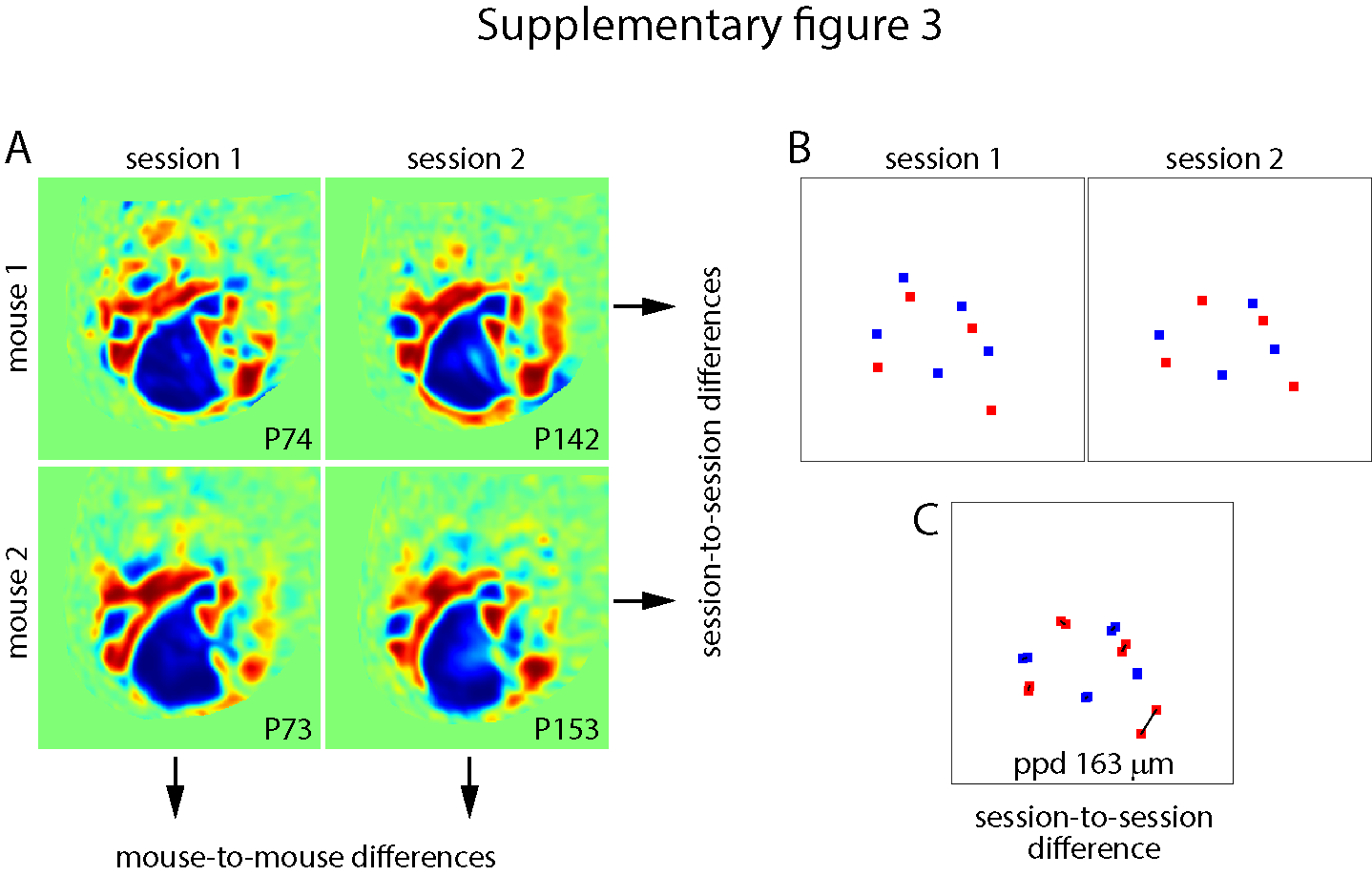

Supplement: S3 Fig — (A) Field sign maps from two mice. For each map, the postnatal age (in days) during imaging is provided. Comparison across mice (across rows) describes the sum of biological variation and measurement noise. Comparison across imaging sessions (down columns) describes measurement noise. The difference between the two comparisons provides an estimate of biological variation. (B) Maps of centroid locations for each field sign patch in mouse 1. Each centroid is colored to match the field sign of its parent field sign patch. (C) Comparison of two maps, with the distances between patches illustrated with black lines. The paired patch distance (ppd) is the mean of these distances. (TIF) [file pone.0213924.s003.tif]

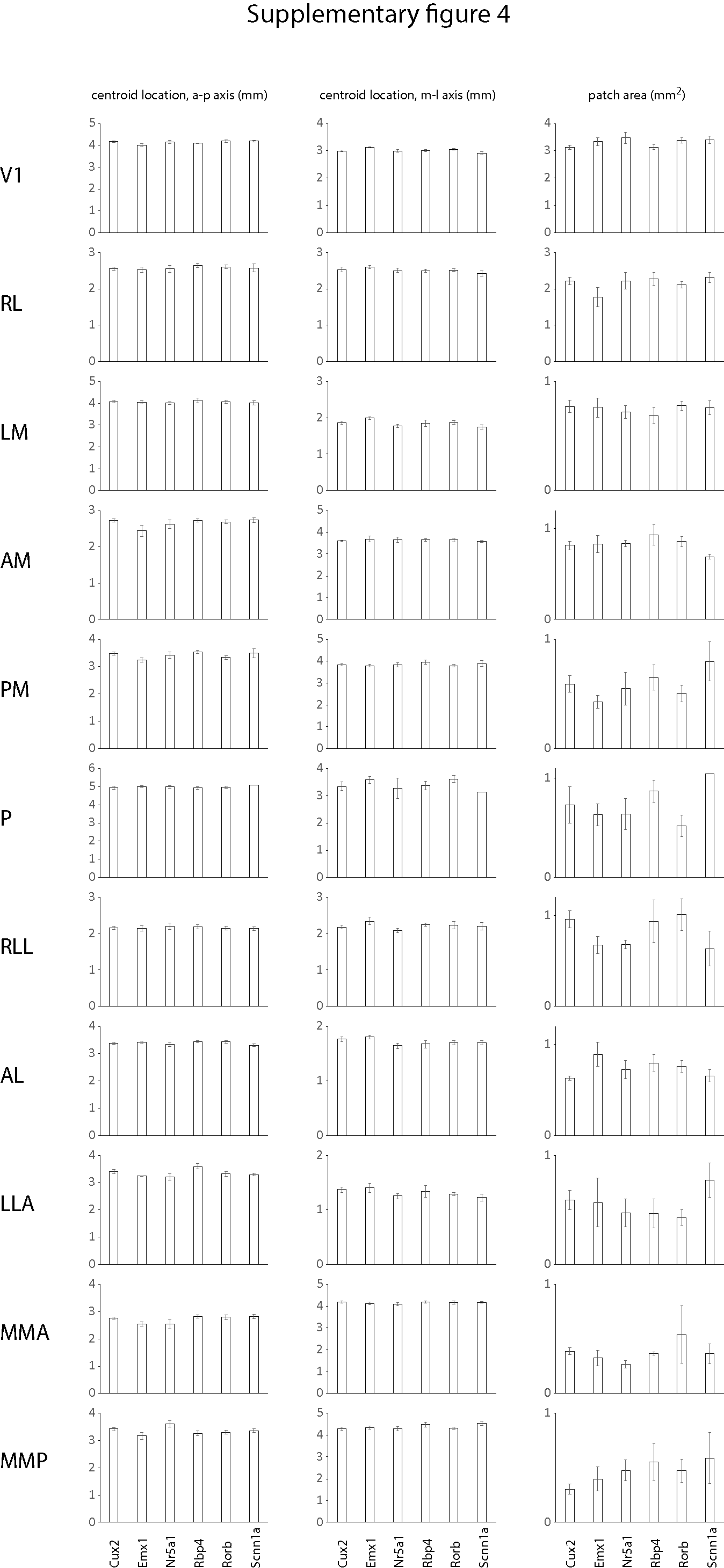

Supplement: S4 Fig — Centroid positions (a-p axis, left column; m-l axis, central column) and surface area (right column) for each patch, sorted by Cre line. No significant differences between Cre lines in any plot (ANOVA, p > 0.05). (TIF) [file pone.0213924.s004.tif]
