# Supplementary material for: Biological variation in the sizes, shapes and locations of visual cortical areas in the mouse
Source: PLoS One. 2019 May 1;14(5):e0213924. doi: 10.1371/journal.pone.0213924 (PMC6493719; doi:10.1371/journal.pone.0213924)
Supplement: S2 Code.html — (HTML) [file pone.0213924.s006.html]

 
 
  
 notebook_data_viewer  
 

 
 
 


 

 
 

 
 
     
     
     
      
 
   
     

 
 
 In [1]: 
 
     
     import   numpy   as   np 
 import   matplotlib.pyplot   as   plt 
 import   datetime 
 import   requests 
 from   six   import   BytesIO 
 from   mpl_toolkits.axes_grid1   import   make_axes_locatable 

 %  matplotlib  inline
 %  load_ext  autoreload
 %  autoreload  2

 print   datetime  .  datetime  .  now  () 
  

 
 
 

 
 


 

  


 
 2019-01-24 12:26:48.965000
 
 
 

 
 

 
 
 
 In [2]: 
 
     
     WKF_URL   =   "http://api.brain-map.org/api/v2/well_known_file_download/{}" 

 def   numpy_load_wkf  (  wkf_id  ,   url  =  WKF_URL  ): 
     url   =   url  .  format  (  wkf_id  ) 
     r   =   requests  .  get  (  url  ) 
     if   r  .  status_code   !=   200  : 
         print  (  "Error retrieving file from {}"  .  format  (  url  )) 
     else  : 
         return   np  .  load  (  BytesIO  (  r  .  content  )) 
    
 # read aligned data sets 
 ISI1_vasculature_stack   =   numpy_load_wkf  (  745546199  ) 
 ISI1_sign_map_stack   =   numpy_load_wkf  (  745546072  ) 
 ISI1_azimuth_map_stack   =   numpy_load_wkf  (  745545159  ) 
 ISI1_altitude_map_stack   =   numpy_load_wkf  (  745544088  ) 
 ISI2_vasculature_stack   =   numpy_load_wkf  (  745597087  ) 
 ISI2_sign_map_stack   =   numpy_load_wkf  (  745596441  ) 
 ISI2_azimuth_map_stack   =   numpy_load_wkf  (  745596342  ) 
 ISI2_altitude_map_stack   =   numpy_load_wkf  (  745546232  ) 
  

 
 
 

 
 
 
 In [3]: 
 
     
     ISI1_list   =   [ 
     '541622887'  , 
     '512415468'  , 
     '531337417'  , 
     '531338127'  , 
     '542117164'  , 
     '542112455'  , 
     '541520741'  , 
     '535676912'  , 
     '540992447'  , 
     '541521621'  , 
     '541621635'  , 
     '543024492'  , 
     '541623655'  , 
     '542269558'  , 
     '542118621'  , 
     '543025312'  , 
     '542267920'  , 
     '542116435'  , 
     '541626956'  , 
     '543018195'  , 
     '539320230'  , 
     '542268849'  , 
     '542266505'  , 
     '542267213'  , 
     '540986357'  , 
     '504720222'  , 
     '505121458'  , 
     '509427495'  , 
     '509435130'  , 
     '509591479'  , 
     '509592187'  , 
     '510654077'  , 
     '511438680'  , 
     '509844206'  , 
     '509842724'  , 
     '512319446'  , 
     '511439388'  , 
     '511559662'  , 
     '511558930'  , 
     '511587340'  , 
     '511940682'  , 
     '512416186'  , 
     '511816486'  , 
     '513042927'  , 
     '511817198'  , 
     '512404385'  , 
     '513244249'  , 
     '513245031'  , 
     '513045901'  , 
     '513486087'  , 
     '513045193'  , 
     '514020745'  , 
     '513245748'  , 
     '514070360'  , 
     '513246501'  , 
     '513247246'  , 
     '528692773'  , 
     '513489967'  , 
     '513044476'  , 
     '539641847'  ] 
  

 
 
 

 
 
 
 In [4]: 
 
     
     ISI2_list   =   [ 
     '514345246'  , 
     '501139891'  , 
     '515910155'  , 
     '515912950'  , 
     '515996070'  , 
     '516024042'  , 
     '517649990'  , 
     '517650091'  , 
     '518632163'  , 
     '518632289'  , 
     '518640920'  , 
     '518756547'  , 
     '519747276'  , 
     '519772020'  , 
     '529204468'  , 
     '521013376'  , 
     '521222627'  , 
     '521948687'  , 
     '522150294'  , 
     '522800444'  , 
     '523504560'  , 
     '524295095'  , 
     '526212914'  , 
     '527109693'  , 
     '521006641'  , 
     '491604967'  , 
     '491604983'  , 
     '491618855'  , 
     '492413952'  , 
     '495726993'  , 
     '495727000'  , 
     '495727015'  , 
     '495727026'  , 
     '496001353'  , 
     '496001358'  , 
     '496144063'  , 
     '497258296'  , 
     '497258332'  , 
     '497589976'  , 
     '501228870'  , 
     '501279658'  , 
     '501281221'  , 
     '501281403'  , 
     '501751610'  , 
     '501753157'  , 
     '501800291'  , 
     '501800347'  , 
     '501800590'  , 
     '502185555'  , 
     '502185594'  , 
     '502206284'  , 
     '503292421'  , 
     '503292439'  , 
     '503292442'  , 
     '503292470'  , 
     '503292481'  , 
     '504298336'  , 
     '504614281'  , 
     '505143644'  , 
     '509522655'  ] 
  

 
 
 

 
 
 
 In [5]: 
 
     
     # view ISI1 images 
 for   ii   in   range  (  len  (  ISI1_list  )): 
     fig   =   plt  .  figure  (  figsize  =  (  20  ,  20  )) 
     fig  .  suptitle  ((  ISI1_list  [  ii  ]),   fontsize  =  12  ) 
    
     ax1   =   fig  .  add_subplot  (  441  ) 
     im1   =   ax1  .  imshow  (  ISI1_vasculature_stack  [  ii  ,:,:],   interpolation  =  'nearest'  ,   cmap  =  'gray'  ) 
     ax1  .  set_axis_off  () 
    
     ax2   =   fig  .  add_subplot  (  442  ) 
     im2   =   ax2  .  imshow  (  ISI1_azimuth_map_stack  [  ii  ,:,:],   interpolation  =  'nearest'  ,   cmap  =  'jet'  ,   clim  =  (  -  30  ,  90  )) 
     ax2  .  set_axis_off  () 
    
     divider   =   make_axes_locatable  (  ax2  ) 
     cax   =   divider  .  append_axes  (  'bottom'  ,   size  =  '5%'  ,   pad  =  0.05  ) 
     fig  .  colorbar  (  im2  ,   cax  =  cax  ,   orientation  =  'horizontal'  ,   ticks  =  [  -  30  ,   0  ,   30  ,   60  ,   90  ],   label  =  'azimuth'  ) 
    
     ax3   =   fig  .  add_subplot  (  443  ) 
     im3   =   ax3  .  imshow  (  ISI1_altitude_map_stack  [  ii  ,:,:],   interpolation  =  'nearest'  ,   cmap  =  'jet'  ,   clim  =  (  -  30  ,  30  )) 
     ax3  .  set_axis_off  () 
    
     divider   =   make_axes_locatable  (  ax3  ) 
     cax   =   divider  .  append_axes  (  'right'  ,   size  =  '5%'  ,   pad  =  0.05  ) 
     fig  .  colorbar  (  im3  ,   cax  =  cax  ,   orientation  =  'vertical'  ,   ticks  =  [  -  30  ,   0  ,   30  ],   label  =  'altitude'  ) 
    
     ax4   =   fig  .  add_subplot  (  444  ) 
     im4   =   ax4  .  imshow  (  ISI1_sign_map_stack  [  ii  ,:,:],   interpolation  =  'nearest'  ,   cmap  =  'jet'  ,   clim  =  (  -  1  ,  1  )) 
     ax4  .  set_axis_off  () 
    
     divider   =   make_axes_locatable  (  ax4  ) 
     cax   =   divider  .  append_axes  (  'right'  ,   size  =  '5%'  ,   pad  =  0.05  ) 
     fig  .  colorbar  (  im4  ,   cax  =  cax  ,   orientation  =  'vertical'  ,   ticks  =  [  -  1  ,   0  ,   1  ]) 
    
     plt  .  show  () 
  

 
 
 

 
 


 

  


 
 
 

 

 

  


 
 
 

 

 

  


 
 
 

 

 

  


 
 
 

 

 

  


 
 
 

 

 

  


 
 
 

 

 

  


 
 
 

 

 

  


 
 
 

 

 

  


 
 
 

 

 

  


 
 
 

 

 

  


 
 
 

 

 

  


 
 
 

 

 

  


 
 
 

 

 

  


 
 
 

 

 

  


 
 
 

 

 

  


 
 
 

 

 

  


 
 
 

 

 

  


 
 
 

 

 

  


 
 
 

 

 

  


 
 
 

 

 

  


 
 
 

 

 

  


 
 
 

 

 

  


 
 
 

 

 

  


 
 
 

 

 

  


 
 
 

 

 

  


 
 
 

 

 

  


 
 
 

 

 

  


 
 
 

 

 

  


 
 
 

 

 

  


 
 
 

 

 

  


 
 
 

 

 

  


 
 
 

 

 

  


 
 
 

 

 

  


 
 
 

 

 

  


 
 
 

 

 

  


 
 
 

 

 

  


 
 
 

 

 

  


 
 
 

 

 

  


 
 
 

 

 

  


 
 
 

 

 

  


 
 
 

 

 

  


 
 
 

 

 

  


 
 
 

 

 

  


 
 
 

 

 

  


 
 
 

 

 

  


 
 
 

 

 

  


 
 
 

 

 

  


 
 
 

 

 

  


 
 
 

 

 

  


 
 
 

 

 

  


 
 
 

 

 

  


 
 
 

 

 

  


 
 
 

 

 

  


 
 
 

 

 

  


 
 
 

 

 

  


 
 
 

 

 

  


 
 
 

 

 

  


 
 
 

 

 

  


 
 
 

 

 

  


 
 
 

 

 
 

 
 
 
 In [6]: 
 
     
     # view ISI2 images 
 for   ii   in   range  (  len  (  ISI2_list  )): 
     fig   =   plt  .  figure  (  figsize  =  (  20  ,  20  )) 
     fig  .  suptitle  ((  ISI2_list  [  ii  ]),   fontsize  =  12  ) 
    
     ax1   =   fig  .  add_subplot  (  441  ) 
     im1   =   ax1  .  imshow  (  ISI2_vasculature_stack  [  ii  ,:,:],   interpolation  =  'nearest'  ,   cmap  =  'gray'  ) 
     ax1  .  set_axis_off  () 
    
     ax2   =   fig  .  add_subplot  (  442  ) 
     im2   =   ax2  .  imshow  (  ISI2_azimuth_map_stack  [  ii  ,:,:],   interpolation  =  'nearest'  ,   cmap  =  'jet'  ,   clim  =  (  -  30  ,  90  )) 
     ax2  .  set_axis_off  () 
    
     divider   =   make_axes_locatable  (  ax2  ) 
     cax   =   divider  .  append_axes  (  'bottom'  ,   size  =  '5%'  ,   pad  =  0.05  ) 
     fig  .  colorbar  (  im2  ,   cax  =  cax  ,   orientation  =  'horizontal'  ,   ticks  =  [  -  30  ,   0  ,   30  ,   60  ,   90  ],   label  =  'azimuth'  ) 
    
     ax3   =   fig  .  add_subplot  (  443  ) 
     im3   =   ax3  .  imshow  (  ISI2_altitude_map_stack  [  ii  ,:,:],   interpolation  =  'nearest'  ,   cmap  =  'jet'  ,   clim  =  (  -  30  ,  30  )) 
     ax3  .  set_axis_off  () 
    
     divider   =   make_axes_locatable  (  ax3  ) 
     cax   =   divider  .  append_axes  (  'right'  ,   size  =  '5%'  ,   pad  =  0.05  ) 
     fig  .  colorbar  (  im3  ,   cax  =  cax  ,   orientation  =  'vertical'  ,   ticks  =  [  -  30  ,   0  ,   30  ],   label  =  'altitude'  ) 
    
     ax4   =   fig  .  add_subplot  (  444  ) 
     im4   =   ax4  .  imshow  (  ISI2_sign_map_stack  [  ii  ,:,:],   interpolation  =  'nearest'  ,   cmap  =  'jet'  ,   clim  =  (  -  1  ,  1  )) 
     ax4  .  set_axis_off  () 
    
     divider   =   make_axes_locatable  (  ax4  ) 
     cax   =   divider  .  append_axes  (  'right'  ,   size  =  '5%'  ,   pad  =  0.05  ) 
     fig  .  colorbar  (  im4  ,   cax  =  cax  ,   orientation  =  'vertical'  ,   ticks  =  [  -  1  ,   0  ,   1  ]) 
    
     plt  .  show  () 
  

 
 
 

 
 


 

  


 
 
 

 

 

  


 
 
 

 

 

  


 
 
 

 

 

  


 
 
 

 

 

  


 
 
 

 

 

  


 
 
 

 

 

  


 
 
 

 

 

  


 
 
 

 

 

  


 
 
 

 

 

  


 
 
 

 

 

  


 
 
 

 

 

  


 
 
 

 

 

  


 
 
 

 

 

  


 
 
 

 

 

  


 
 
 

 

 

  


 
 
 

 

 

  


 
 
 

 

 

  


 
 
 

 

 

  


 
 
 

 

 

  


 
 
 

 

 

  


 
 
 

 

 

  


 
 
 

 

 

  


 
 
 

 

 

  


 
 
 

 

 

  


 
 
 

 

 

  


 
 
 

 

 

  


 
 
 

 

 

  


 
 
 

 

 

  


 
 
 

 

 

  


 
 
 

 

 

  


 
 
 

 

 

  


 
 
 

 

 

  


 
 
 

 

 

  


 
 
 

 

 

  


 
 
 

 

 

  


 
 
 

 

 

  


 
 
 

 

 

  


 
 
 

 

 

  


 
 
 

 

 

  


 
 
 

 

 

  


 
 
 

 

 

  


 
 
 

 

 

  


 
 
 

 

 

  


 
 
 

 

 

  


 
 
 

 

 

  


 
 
 

 

 

  


 
 
 

 

 

  


 
 
 

 

 

  


 
 
 

 

 

  


 
 
 

 

 

  


 
 
 

 

 

  


 
 
 

 

 

  


 
 
 

 

 

  


 
 
 

 

 

  


 
 
 

 

 

  


 
 
 

 

 

  


 
 
 

 

 

  


 
 
 

 

 

  


 
 
 

 

 

  


 
 
 

 

 
 

 
 
 
 In [7]: 
 
     
     # check alignment of ISI1 and ISI2 maps 

 for   ii   in   range  (  len  (  ISI1_list  )): 
     fig   =   plt  .  figure  (  figsize  =  (  10  ,  10  )) 
     fig  .  suptitle  ((  ISI1_list  [  ii  ],  ISI2_list  [  ii  ]),   fontsize  =  12  ) 
    
     ax1   =   fig  .  add_subplot  (  221  ) 
     ax1  .  imshow  (  ISI1_vasculature_stack  [  ii  ,:,:],   interpolation  =  'nearest'  ,   cmap  =  'gray'  ) 
     ax1  .  imshow  (  ISI2_vasculature_stack  [  ii  ,:,:],   interpolation  =  'nearest'  ,   cmap  =  'gray'  ,   alpha   =   0.5  ) 
     ax1  .  set_axis_off  () 
    
     ax2   =   fig  .  add_subplot  (  222  ) 
     ax2  .  imshow  (  ISI1_sign_map_stack  [  ii  ,:,:],   interpolation  =  'nearest'  ,   cmap  =  'jet'  ) 
     ax2  .  imshow  (  ISI2_sign_map_stack  [  ii  ,:,:],   interpolation  =  'nearest'  ,   cmap  =  'jet'  ,   alpha   =   0.5  ) 
     ax2  .  set_axis_off  () 
        
     plt  .  show  () 
  

 
 
 

 
 


 

  


 
 
 

 

 

  


 
 
 

 

 

  


 
 
 

 

 

  


 
 
 

 

 

  


 
 
 

 

 

  


 
 
 

 

 

  


 
 
 

 

 

  


 
 
 

 

 

  


 
 
 

 

 

  


 
 
 

 

 

  


 
 
 

 

 

  


 
 
 

 

 

  


 
 
 

 

 

  


 
 
 

 

 

  


 
 
 

 

 

  


 
 
 

 

 

  


 
 
 

 

 

  


 
 
 

 

 

  


 
 
 

 

 

  


 
 
 

 

 

  


 
 
 

 

 

  


 
 
 

 

 

  


 
 
 

 

 

  


 
 
 

 

 

  


 
 
 

 

 

  


 
 
 

 

 

  


 
 
 

 

 

  


 
 
 

 

 

  


 
 
 

 

 

  


 
 
 

 

 

  


 
 
 

 

 

  


 
 
 

 

 

  


 
 
 

 

 

  


 
 
 

 

 

  


 
 
 

 

 

  


 
 
 

 

 

  


 
 
 

 

 

  


 
 
 

 

 

  


 
 
 

 

 

  


 
 
 

 

 

  


 
 
 

 

 

  


 
 
 

 

 

  


 
 
 

 

 

  


 
 
 

 

 

  


 
 
 

 

 

  


 
 
 

 

 

  


 
 
 

 

 

  


 
 
 

 

 

  


 
 
 

 

 

  


 
 
 

 

 

  


 
 
 

 

 

  


 
 
 

 

 

  


 
 
 

 

 

  


 
 
 

 

 

  


 
 
 

 

 

  


 
 
 

 

 

  


 
 
 

 

 

  


 
 
 

 

 

  


 
 
 

 

 

  


 
 
 

 

 
 

 
     
   
 

 


 
