# Supplementary material for: Biological variation in the sizes, shapes and locations of visual cortical areas in the mouse
Source: PLoS One. 2019 May 1;14(5):e0213924. doi: 10.1371/journal.pone.0213924 (PMC6493719; doi:10.1371/journal.pone.0213924)
Supplement: S4 Code.html — (HTML) [file pone.0213924.s008.html]

notebook\_analysis


# 1. Imports¶

In [1]:

```
import numpy as np
import matplotlib.pyplot as plt
import matplotlib.mlab as mlab
import scipy.ndimage.morphology
import scipy.stats
import cv2
import datetime
from IPython.display import clear_output
import requests
from six import BytesIO
import tifffile as tiff
import retinotopic_mapping.RetinotopicMapping as rm # available at https://github.com/zhuangjun1981/retinotopic_mapping

%matplotlib inline
%load_ext autoreload
%autoreload 2

print datetime.datetime.now()
```

```
2019-01-24 12:26:55.845000
```

In [2]:

```
p_criterion = 0.05
```

# 2. Read data sets¶

In [3]:

```
WKF_URL = "http://api.brain-map.org/api/v2/well_known_file_download/{}"

def numpy_load_wkf(wkf_id, url=WKF_URL):
    url = url.format(wkf_id)
    r = requests.get(url)
    if r.status_code != 200:
        print("Error retrieving file from {}".format(url))
    else:
        return np.load(BytesIO(r.content))
    
# read aligned data sets
ISI1_vasculature_stack = numpy_load_wkf(745546199)
ISI1_sign_map_stack = numpy_load_wkf(745546072)
ISI1_azimuth_map_stack = numpy_load_wkf(745545159)
ISI1_altitude_map_stack = numpy_load_wkf(745544088)
ISI2_vasculature_stack = numpy_load_wkf(745597087)
ISI2_sign_map_stack = numpy_load_wkf(745596441)
ISI2_azimuth_map_stack = numpy_load_wkf(745596342)
ISI2_altitude_map_stack = numpy_load_wkf(745546232)
```

In [4]:

```
ISI1_list = [
    '541622887',
    '512415468',
    '531337417',
    '531338127',
    '542117164',
    '542112455',
    '541520741',
    '535676912',
    '540992447',
    '541521621',
    '541621635',
    '543024492',
    '541623655',
    '542269558',
    '542118621',
    '543025312',
    '542267920',
    '542116435',
    '541626956',
    '543018195',
    '539320230',
    '542268849',
    '542266505',
    '542267213',
    '540986357',
    '504720222',
    '505121458',
    '509427495',
    '509435130',
    '509591479',
    '509592187',
    '510654077',
    '511438680',
    '509844206',
    '509842724',
    '512319446',
    '511439388',
    '511559662',
    '511558930',
    '511587340',
    '511940682',
    '512416186',
    '511816486',
    '513042927',
    '511817198',
    '512404385',
    '513244249',
    '513245031',
    '513045901',
    '513486087',
    '513045193',
    '514020745',
    '513245748',
    '514070360',
    '513246501',
    '513247246',
    '528692773',
    '513489967',
    '513044476',
    '539641847']
```

In [5]:

```
ISI2_list = [
    '514345246',
    '501139891',
    '515910155',
    '515912950',
    '515996070',
    '516024042',
    '517649990',
    '517650091',
    '518632163',
    '518632289',
    '518640920',
    '518756547',
    '519747276',
    '519772020',
    '529204468',
    '521013376',
    '521222627',
    '521948687',
    '522150294',
    '522800444',
    '523504560',
    '524295095',
    '526212914',
    '527109693',
    '521006641',
    '491604967',
    '491604983',
    '491618855',
    '492413952',
    '495726993',
    '495727000',
    '495727015',
    '495727026',
    '496001353',
    '496001358',
    '496144063',
    '497258296',
    '497258332',
    '497589976',
    '501228870',
    '501279658',
    '501281221',
    '501281403',
    '501751610',
    '501753157',
    '501800291',
    '501800347',
    '501800590',
    '502185555',
    '502185594',
    '502206284',
    '503292421',
    '503292439',
    '503292442',
    '503292470',
    '503292481',
    '504298336',
    '504614281',
    '505143644',
    '509522655']
```

# 3. Segment and identify patches¶

## 3.1 Calculate mean map from ISI1¶

In [6]:

```
mean_altitude_map = np.copy(ISI1_altitude_map_stack)
mean_azimuth_map = np.copy(ISI1_azimuth_map_stack)

for ii in range(len(ISI1_list)):
    altitude_map = mean_altitude_map[ii,:,:]
    azimuth_map = mean_azimuth_map[ii,:,:]
    
    # convert all values with no data to NaNs
    altitude_map[altitude_map == altitude_map[0,0]] = np.nan
    azimuth_map[azimuth_map == azimuth_map[0,0]] = np.nan
    
    mean_altitude_map[ii,:,:] = altitude_map
    mean_azimuth_map[ii,:,:] = azimuth_map

mean_altitude_map = np.nanmean(mean_altitude_map, axis=0)
mean_azimuth_map = np.nanmean(mean_azimuth_map, axis=0)
```

```
C:\anaconda2\lib\site-packages\ipykernel_launcher.py:15: RuntimeWarning: Mean of empty slice
  from ipykernel import kernelapp as app
C:\anaconda2\lib\site-packages\ipykernel_launcher.py:16: RuntimeWarning: Mean of empty slice
  app.launch_new_instance()
```

In [ ]:

```
# make empty mask to substitute for missing maps
empty_mask = np.zeros_like(ISI1_vasculature_stack[0])

# remove nans before calculating sign maps
mean_altitude_map[np.isnan(mean_altitude_map)] = 1000
mean_azimuth_map[np.isnan(mean_azimuth_map)] = 1000

# make sign and patch maps
params = {
          'phaseMapFilterSigma': 0.5,
          'signMapFilterSigma': 8.,
          'signMapThr': 0.23,
          'eccMapFilterSigma': 15.0,
          'splitLocalMinCutStep': 5.,
          'closeIter': 3,
          'openIter': 3,
          'dilationIter': 15,
          'borderWidth': 1,
          'smallPatchThr': 100,
          'visualSpacePixelSize': 0.5,
          'visualSpaceCloseIter': 15,
          'splitOverlapThr': 1.1,
          'mergeOverlapThr': 0.1
          }

trial = rm.RetinotopicMappingTrial(altPosMap=mean_altitude_map,
                                   aziPosMap=mean_azimuth_map,
                                   altPowerMap=np.amax(empty_mask, axis=1),
                                   aziPowerMap=np.amax(empty_mask, axis=1),
                                   vasculatureMap=np.amax(empty_mask, axis=1),
                                   mouseID='test',
                                   dateRecorded='111111',
                                   comments='No comment.',
                                   params=params)

_ = trial._getSignMap(isPlot=False, isReverse=False, isFixedRange=False)
_ = trial._getRawPatches(isPlot=False)
_ = trial._getDeterminantMap(isPlot=False)
_ = trial._getEccentricityMap(isPlot=False)
_ = trial._splitPatches(isPlot=False)
_ = trial._mergePatches(isPlot=False)

clear_output()
```

In [8]:

```
plt.show()
```

In [9]:

```
signmap = trial.signMapf
```

In [10]:

```
border_mask = np.zeros(mean_altitude_map.shape)

for ii in range(len(trial.finalPatches.keys())):
    area_mask = trial.finalPatches[trial.finalPatches.keys()[ii]].array
    border_mask = border_mask + area_mask - scipy.ndimage.morphology.binary_erosion(area_mask, iterations=1)

fig = plt.figure(figsize=(7,7))
ax1 = fig.add_subplot(111)
ax1.imshow(signmap + 10*border_mask, clim=(-1,1), cmap='jet')
plt.show()
```

In [11]:

```
ISI1_mean_signmap = signmap
ISI1_borders = border_mask
```

## 3.2 Make template from which to identify patches in each map¶

In [12]:

```
# template based on ISI1 map
# view segmented ISI1 map
print len(trial.finalPatches.keys()), 'patches in mean sign map'

area_sum = np.zeros(area_mask.shape)
for ii in range(len(trial.finalPatches.keys())):
    area_mask = trial.finalPatches[trial.finalPatches.keys()[ii]].array
    area_sum = area_sum + area_mask * (ii + 1)
plt.imshow(area_sum, cmap='jet')
plt.colorbar()
plt.show()
```

```
16 patches in mean sign map
```

display = np.copy(area\_sum)
display[area\_sum != 4] = 0
plt.imshow(display, clim=(0,16))# manually identify patches
0 discard
1 AL
2 discard
3 discard
4 discard
5 discard
6 MMA
7 AM
8 LLA
9 MMP
10 LM
11 discard
12 discard
13 V1
14 RLL
15 P & PM - need to separate manually
16 RL

In [13]:

```
# make list of patches, in order to search for them
# make corresponding field sign list
# make corresponding set of masks

mean_area_name_list = ['V1','RL','LM','AM','PM','P','RLL','AL','LLA','MMA','MMP']

mean_field_sign_list = [-1,1,1,-1,1,1,-1,-1,1,1,-1]

mean_mask_list = [trial.finalPatches[trial.finalPatches.keys()[12]].array,
                  trial.finalPatches[trial.finalPatches.keys()[15]].array,
                  trial.finalPatches[trial.finalPatches.keys()[9]].array,
                  trial.finalPatches[trial.finalPatches.keys()[6]].array,
                  trial.finalPatches[trial.finalPatches.keys()[14]].array, # PM
                  trial.finalPatches[trial.finalPatches.keys()[14]].array, # P
                  trial.finalPatches[trial.finalPatches.keys()[13]].array,
                  trial.finalPatches[trial.finalPatches.keys()[0]].array,
                  trial.finalPatches[trial.finalPatches.keys()[7]].array,
                  trial.finalPatches[trial.finalPatches.keys()[5]].array,
                  trial.finalPatches[trial.finalPatches.keys()[8]].array,
                 ]

#split P and PM
mean_mask_list[4][420:,:] = 0
mean_mask_list[4][380:600,400:600] = 0
mean_mask_list[5][0:480,:] = 0
mean_mask_list[5][:,400:] = 0

# make centroid array (in pixels)
mean_ISI1_centroid_array = np.zeros((len(mean_mask_list),2))
for ii in range(len(mean_mask_list)):
    mean_ISI1_centroid_array[ii,0] = scipy.ndimage.measurements.center_of_mass(mean_mask_list[ii])[0]
    mean_ISI1_centroid_array[ii,1] = scipy.ndimage.measurements.center_of_mass(mean_mask_list[ii])[1]
```

In [14]:

```
# recalculate area_sum without discarded patches

area_sum = np.zeros(mean_mask_list[0].shape)
for ii in range(len(mean_mask_list)):
    if (mean_area_name_list[ii] != 'discard'):
        area_sum = area_sum + mean_mask_list[ii] * (ii + 1)

fig = plt.figure(figsize=(7,7))
ax1 = fig.add_subplot(111)
ax1.imshow(area_sum, cmap='jet')
for ii in range(len(mean_area_name_list)):
    ax1.text(mean_ISI1_centroid_array[ii,1], mean_ISI1_centroid_array[ii,0], mean_area_name_list[ii], fontsize=10, color='white')
plt.show()
```

In [15]:

```
# calculate maximum extent and area of each patch
for ii in range(len(mean_mask_list)):
    patch = mean_mask_list[ii]
    
    x_dim = 0.
    y_dim = 0.
    for jj in range(600):
        if np.sum(patch[jj,:]) > x_dim:
            x_dim = np.sum(patch[jj,:])
        if np.sum(patch[:,jj]) > y_dim:
            y_dim = np.sum(patch[:,jj])

    # convert to mm
    x_dim = x_dim / 100.
    y_dim = y_dim / 100.

    print mean_area_name_list[ii], ', x dim', x_dim, ', y dim', y_dim, ' mm.'
    print mean_area_name_list[ii], 'area in mm2', np.count_nonzero(mean_mask_list[ii]) / 10000.
```

```
V1 , x dim 1.81 , y dim 2.23  mm.
V1 area in mm2 2.8281
RL , x dim 2.0 , y dim 1.28  mm.
RL area in mm2 2.1502
LM , x dim 0.83 , y dim 1.25  mm.
LM area in mm2 0.6977
AM , x dim 0.86 , y dim 0.63  mm.
AM area in mm2 0.3943
PM , x dim 0.59 , y dim 1.15  mm.
PM area in mm2 0.4461
P , x dim 1.84 , y dim 0.45  mm.
P area in mm2 0.5956
RLL , x dim 0.82 , y dim 0.58  mm.
RLL area in mm2 0.3583
AL , x dim 0.58 , y dim 0.68  mm.
AL area in mm2 0.3438
LLA , x dim 0.45 , y dim 0.87  mm.
LLA area in mm2 0.275
MMA , x dim 0.61 , y dim 0.79  mm.
MMA area in mm2 0.382
MMP , x dim 0.77 , y dim 1.0  mm.
MMP area in mm2 0.6115
```

## 3.3 Identify and log patches¶

In [16]:

```
params = {
          'phaseMapFilterSigma': 0.5,
          'signMapFilterSigma': 10.,
          'signMapThr': 0.25,
          'eccMapFilterSigma': 15.0,
          'splitLocalMinCutStep': 5.,
          'closeIter': 3,
          'openIter': 3,
          'dilationIter': 15,
          'borderWidth': 1,
          'smallPatchThr': 100,
          'visualSpacePixelSize': 0.5,
          'visualSpaceCloseIter': 15,
          'splitOverlapThr': 1.1,
          'mergeOverlapThr': 0.1
          }
```

### 3.3.1 Imaging session 1¶

In [ ]:

```
# make lists containing sign maps and segmented sign maps for all the experiments in ISI1_list
ISI1_signmap_list = []
ISI1_signmap_seg_list = []

for ii in range(len(ISI1_list)):
    print 'iterations to completion:', len(ISI1_list)
    print 'current iteration', ii
    
    # generate borders for ISI1
    trial = rm.RetinotopicMappingTrial(altPosMap=ISI1_altitude_map_stack[ii,:,:],
                                   aziPosMap=ISI1_azimuth_map_stack[ii,:,:],
                                   altPowerMap=np.amax(empty_mask, axis=1),
                                   aziPowerMap=np.amax(empty_mask, axis=1),
                                   vasculatureMap=np.amax(empty_mask, axis=1),
                                   mouseID='test',
                                   dateRecorded='111111',
                                   comments='No comment.',
                                   params=params)
    
    _ = trial._getSignMap(isPlot=False, isReverse=False, isFixedRange=False)
    _ = trial._getRawPatches(isPlot=False)
    _ = trial._getDeterminantMap(isPlot=False)
    _ = trial._getEccentricityMap(isPlot=False)
    _ = trial._splitPatches(isPlot=False)
    _ = trial._mergePatches(isPlot=False)
    clear_output()
    
    # add results to lists
    ISI1_signmap_list.append(trial.signMapf)
    ISI1_signmap_seg = np.zeros(trial.signMapf.shape)
    for jj in range(len(trial.finalPatches.keys())):
        area_mask = trial.finalPatches[trial.finalPatches.keys()[jj]].array
        ISI1_signmap_seg[area_mask == 1] = jj + 1
    ISI1_signmap_seg_list.append(ISI1_signmap_seg)
```

In [18]:

```
plt.show()
```

In [19]:

```
# for ISI1, assign names to each patch in the segmented sign map

# first, make a list with 60 entries, each with 11 entries, each a binary mask (containing zeros)
# for each patch in mean list, calculate intersection/union ratio for all patches with same field sign
# and assign label to patch with largest intersection/union ratio, removing this patch from the list
# rinse, repeat through 11 patches in mean patch map (11 iterations of the loop)
# if no patch has any overlap, leave this map blank (zeros)

# RL and P are handled a little differently, since they're being cut into sub-regions.
# For RL and P, we calculate Jaccard Index for all patches with positive field sign and assign to RL and P all
# patches with Jaccard Index greater than the threshold value, i.e. more than one patch can be amalgamated to
# form RL and P.

overlap_threshold = 0.1

ISI1_patch_mask_list = []

list_of_patches_ISI1 = []
# iterate through experiments making lists of field signs and masks for each experiment
for ii in range(len(ISI1_list)):
    ISI1_mask_list = []
    ISI1_field_sign_list = []
    for jj in range((np.amax(ISI1_signmap_seg_list[ii])).astype(np.int)):    #iterate through segmented patches
        segmented_area = np.zeros(ISI1_signmap_seg_list[ii].shape, dtype=bool)
        segmented_area[ISI1_signmap_seg_list[ii] == jj + 1] = True
        ISI1_mask_list.append(segmented_area)
        ISI1_field_sign_list.append(np.sign(np.mean(ISI1_signmap_list[ii][segmented_area == True])))
    # at this point we have initial lists of field signs and masks for this experiment   
    
    # run through patches (in order, by decreasing surface area) and assign a name to (or discard) each segmented patch
    mask_list = []
    ISI1_mask_list_copy = list(ISI1_mask_list)
    
    # first find V1
    overlap_ratios = np.zeros(len(ISI1_field_sign_list))
    for jj in range(len(ISI1_field_sign_list)):
        # if segmented_area ii has same field sign as mean map area kk...
        if (ISI1_field_sign_list[jj] == -1):
            overlap_ratios[jj] = np.sum((np.logical_and(mean_mask_list[0], ISI1_mask_list_copy[jj]))) / \
                      np.sum((np.logical_or(mean_mask_list[0], ISI1_mask_list_copy[jj]))).astype(np.float)
    mask_list.append(ISI1_mask_list_copy[np.ndarray.tolist(overlap_ratios).index(np.amax(overlap_ratios))])
    ISI1_mask_list_copy[np.ndarray.tolist(overlap_ratios).index(np.amax(overlap_ratios))] = \
                                                                     np.zeros(ISI1_mask_list_copy[0].shape)
    
    # align map to mean map: shift map by difference between V1 centroids
    # V1 mask for this map is mask_list[0]
    # V1 mask for mean map is mean_mask_list[0]
    # difference in centroid position is
    x_shift = scipy.ndimage.measurements.center_of_mass(np.asarray(mean_mask_list[0]))[0] - \
                   scipy.ndimage.measurements.center_of_mass(np.asarray(mask_list[0]))[0]
    y_shift = scipy.ndimage.measurements.center_of_mass(np.asarray(mean_mask_list[0]))[1] - \
                   scipy.ndimage.measurements.center_of_mass(np.asarray(mask_list[0]))[1]
    translation_matrix = np.float32([[1,0,y_shift],[0,1,x_shift]])
    
    ISI1_mask_list_copy = list(ISI1_mask_list)
    for nn in range(len(ISI1_mask_list)):
        ISI1_mask_list_copy[nn] = cv2.warpAffine(ISI1_mask_list[nn].astype(np.float), translation_matrix, \
                                                 ISI1_mask_list[nn].shape).astype(np.bool)
    
    # run through patches (in order, by decreasing surface area) and assign a name to (or discard) each segmented patch
    mask_list = []
    
    # find other areas
    for kk in range(0, len(mean_area_name_list)):
        overlap_ratios = np.zeros(len(ISI1_field_sign_list))
        for jj in range(len(ISI1_field_sign_list)):
            # if segmented_area ii has same field sign as mean map area kk...
            if (mean_field_sign_list[kk] == ISI1_field_sign_list[jj]):
                overlap_ratios[jj] = np.sum((np.logical_and(mean_mask_list[kk], ISI1_mask_list_copy[jj]))) / \
                          np.sum((np.logical_or(mean_mask_list[kk], ISI1_mask_list_copy[jj]))).astype(np.float)        
        if kk == 1 or kk == 5: # if mean patch is RL or P
            if len(np.where(overlap_ratios > overlap_threshold)[0]) > 1:
                mask_temp = np.zeros(segmented_area.shape, dtype=bool)
                for jj in range(len(np.where(overlap_ratios > overlap_threshold)[0])):
                    mask_temp = mask_temp + ISI1_mask_list_copy[np.where(overlap_ratios > overlap_threshold)[0][jj]]
                    ISI1_mask_list_copy[np.where(overlap_ratios > overlap_threshold)[0][jj]] = \
                                                                         np.zeros(ISI1_mask_list_copy[0].shape)
                mask_list.append(mask_temp)
            elif len(np.where(overlap_ratios > overlap_threshold)[0]) == 1:
                mask_list.append(ISI1_mask_list_copy[np.ndarray.tolist(overlap_ratios).index(np.amax(overlap_ratios))])
                ISI1_mask_list_copy[np.ndarray.tolist(overlap_ratios).index(np.amax(overlap_ratios))] = \
                                                                         np.zeros(ISI1_mask_list_copy[0].shape)
            else:
                mask_list.append(np.zeros(segmented_area.shape, dtype=bool))
        else: # if not RL or P
            if np.amax(overlap_ratios) > 0:
                mask_list.append(ISI1_mask_list_copy[np.ndarray.tolist(overlap_ratios).index(np.amax(overlap_ratios))])
            else:
                mask_list.append(np.zeros(segmented_area.shape, dtype=bool))
            ISI1_mask_list_copy[np.ndarray.tolist(overlap_ratios).index(np.amax(overlap_ratios))] = \
                                                                         np.zeros(ISI1_mask_list_copy[0].shape)

    # reverse shift in centroid of each patch, used to identify patches
    translation_matrix = np.float32([[1,0,-y_shift],[0,1,-x_shift]])
    for nn in range(len(mask_list)):
        mask_list[nn] = cv2.warpAffine(mask_list[nn].astype(np.float), translation_matrix, \
                                                      mask_list[nn].shape).astype(np.bool)
    
    # add ordered list of masks to ISI1_patch_mask_list
    ISI1_patch_mask_list.append(mask_list)
    
    # plot results
    fig = plt.figure(figsize=(12,12))
    fig.suptitle((ISI1_list[ii]), fontsize=12)
    ax1 = fig.add_subplot(221)
    ax1.imshow(ISI1_sign_map_stack[ii], interpolation='nearest', cmap='jet')
    ax2 = fig.add_subplot(222)
    for jj in range(len(ISI1_mask_list)):
        segmented_mask = np.zeros(ISI1_mask_list[jj].shape)
        segmented_mask[:] = np.nan
        segmented_mask[ISI1_mask_list[jj] == True] = 1
        if ISI1_field_sign_list[jj] == -1:
            segmented_mask = segmented_mask * -1
        ax2.imshow(segmented_mask, interpolation='none', alpha=0.5, clim=(-1,1), cmap='jet')
    
    for jj in range(len(mean_field_sign_list)):
        segmented_mask = np.empty(ISI1_sign_map_stack[ii,:,:].shape)
        segmented_mask[:] = np.nan
        segmented_mask[mask_list[jj] == 1] = mean_field_sign_list[jj]
        if np.sum(mask_list[jj]) > 0:
            ax2.imshow(segmented_mask, interpolation='none', clim=(-1,1), cmap='jet')
            ax2.text(scipy.ndimage.measurements.center_of_mass(np.asarray(mask_list)[jj])[1], 
                     scipy.ndimage.measurements.center_of_mass(np.asarray(mask_list)[jj])[0], 
                     mean_area_name_list[jj], fontsize=10, color='white')    
    plt.show()
```

### 3.3.2 Imaging session 2¶

In [ ]:

```
# make lists containing sign maps and segmented sign maps for all the experiments in ISI2_list
ISI2_signmap_list = []
ISI2_signmap_seg_list = []

for ii in range(len(ISI2_list)):
    print 'iterations to completion:', len(ISI2_list)
    print 'current iteration', ii
    
    # generate borders for ISI2
    trial = rm.RetinotopicMappingTrial(altPosMap=ISI2_altitude_map_stack[ii,:,:],
                                   aziPosMap=ISI2_azimuth_map_stack[ii,:,:],
                                   altPowerMap=np.amax(empty_mask, axis=1),
                                   aziPowerMap=np.amax(empty_mask, axis=1),
                                   vasculatureMap=np.amax(empty_mask, axis=1),
                                   mouseID='test',
                                   dateRecorded='111111',
                                   comments='No comment.',
                                   params=params)
    
    _ = trial._getSignMap(isPlot=False, isReverse=False, isFixedRange=False)
    _ = trial._getRawPatches(isPlot=False)
    _ = trial._getDeterminantMap(isPlot=False)
    _ = trial._getEccentricityMap(isPlot=False)
    _ = trial._splitPatches(isPlot=False)
    _ = trial._mergePatches(isPlot=False)
    clear_output()
    
    # add results to lists
    ISI2_signmap_list.append(trial.signMapf)
    ISI2_signmap_seg = np.zeros(trial.signMapf.shape)
    for jj in range(len(trial.finalPatches.keys())):
        area_mask = trial.finalPatches[trial.finalPatches.keys()[jj]].array
        ISI2_signmap_seg[area_mask == 1] = jj + 1
    ISI2_signmap_seg_list.append(ISI2_signmap_seg)
```

In [21]:

```
plt.show()
```

In [22]:

```
# for ISI2, assign names to each patch in the segmented sign map

# first, make a list with 60 entries, each with 11 entries, each a binary mask (containing zeros)
# for each patch in mean list, calculate intersection/union ratio for all patches with same field sign
# and assign label to patch with largest intersection/union ratio, removing this patch from the list
# rinse, repeat through 11 patches in mean patch map (11 iterations of the loop)
# if no patch has any overlap, leave this map blank (zeros)

# RL and P are handled a little differently, since they're being cut into sub-regions.
# For RL and P, we calculate Jaccard Index for all patches with positive field sign and assign to RL and P all
# patches with Jaccard Index greater than the threshold value, i.e. more than one patch can be amalgamated to
# form RL and P.

overlap_threshold = 0.1

ISI2_patch_mask_list = []

list_of_patches_ISI2 = []
# iterate through experiments making lists of field signs and masks for each experiment
for ii in range(len(ISI2_list)):
    ISI2_mask_list = []
    ISI2_field_sign_list = []
    for jj in range((np.amax(ISI2_signmap_seg_list[ii])).astype(np.int)):    #iterate through segmented patches
        segmented_area = np.zeros(ISI2_signmap_seg_list[ii].shape, dtype=bool)
        segmented_area[ISI2_signmap_seg_list[ii] == jj + 1] = True
        ISI2_mask_list.append(segmented_area)
        ISI2_field_sign_list.append(np.sign(np.mean(ISI2_signmap_list[ii][segmented_area == True])))
    # at this point we have initial lists of field signs and masks for this experiment   
    
    # run through patches (in order, by decreasing surface area) and assign a name to (or discard) each segmented patch
    mask_list = []
    ISI2_mask_list_copy = list(ISI2_mask_list)
    
    # first find V1
    overlap_ratios = np.zeros(len(ISI2_field_sign_list))
    for jj in range(len(ISI2_field_sign_list)):
        # if segmented_area ii has same field sign as mean map area kk...
        if (ISI2_field_sign_list[jj] == -1):
            overlap_ratios[jj] = np.sum((np.logical_and(mean_mask_list[0], ISI2_mask_list_copy[jj]))) / \
                      np.sum((np.logical_or(mean_mask_list[0], ISI2_mask_list_copy[jj]))).astype(np.float)
    mask_list.append(ISI2_mask_list_copy[np.ndarray.tolist(overlap_ratios).index(np.amax(overlap_ratios))])
    ISI2_mask_list_copy[np.ndarray.tolist(overlap_ratios).index(np.amax(overlap_ratios))] = \
                                                                     np.zeros(ISI2_mask_list_copy[0].shape)
    
    # align map to mean map: shift map by difference between V1 centroids
    # V1 mask for this map is mask_list[0]
    # V1 mask for mean map is mean_mask_list[0]
    # difference in centroid position is
    x_shift = scipy.ndimage.measurements.center_of_mass(np.asarray(mean_mask_list[0]))[0] - \
                   scipy.ndimage.measurements.center_of_mass(np.asarray(mask_list[0]))[0]
    y_shift = scipy.ndimage.measurements.center_of_mass(np.asarray(mean_mask_list[0]))[1] - \
                   scipy.ndimage.measurements.center_of_mass(np.asarray(mask_list[0]))[1]
    translation_matrix = np.float32([[1,0,y_shift],[0,1,x_shift]])
    
    ISI2_mask_list_copy = list(ISI2_mask_list)
    for nn in range(len(ISI2_mask_list)):
        ISI2_mask_list_copy[nn] = cv2.warpAffine(ISI2_mask_list[nn].astype(np.float), translation_matrix, \
                                                 ISI2_mask_list[nn].shape).astype(np.bool)
    
    # run through patches (in order, by decreasing surface area) and assign a name to (or discard) each segmented patch
    mask_list = []
    
    # find other areas
    for kk in range(0, len(mean_area_name_list)):
        overlap_ratios = np.zeros(len(ISI2_field_sign_list))
        for jj in range(len(ISI2_field_sign_list)):
            # if segmented_area ii has same field sign as mean map area kk...
            if (mean_field_sign_list[kk] == ISI2_field_sign_list[jj]):
                overlap_ratios[jj] = np.sum((np.logical_and(mean_mask_list[kk], ISI2_mask_list_copy[jj]))) / \
                          np.sum((np.logical_or(mean_mask_list[kk], ISI2_mask_list_copy[jj]))).astype(np.float)        
        if kk == 1 or kk == 5: # if mean patch is RL or P
            if len(np.where(overlap_ratios > overlap_threshold)[0]) > 1:
                mask_temp = np.zeros(segmented_area.shape, dtype=bool)
                for jj in range(len(np.where(overlap_ratios > overlap_threshold)[0])):
                    mask_temp = mask_temp + ISI2_mask_list_copy[np.where(overlap_ratios > overlap_threshold)[0][jj]]
                    ISI2_mask_list_copy[np.where(overlap_ratios > overlap_threshold)[0][jj]] = \
                                                                         np.zeros(ISI2_mask_list_copy[0].shape)
                mask_list.append(mask_temp)
            elif len(np.where(overlap_ratios > overlap_threshold)[0]) == 1:
                mask_list.append(ISI2_mask_list_copy[np.ndarray.tolist(overlap_ratios).index(np.amax(overlap_ratios))])
                ISI2_mask_list_copy[np.ndarray.tolist(overlap_ratios).index(np.amax(overlap_ratios))] = \
                                                                         np.zeros(ISI2_mask_list_copy[0].shape)
            else:
                mask_list.append(np.zeros(segmented_area.shape, dtype=bool))
        else: # if not RL or P
            if np.amax(overlap_ratios) > 0:
                mask_list.append(ISI2_mask_list_copy[np.ndarray.tolist(overlap_ratios).index(np.amax(overlap_ratios))])
            else:
                mask_list.append(np.zeros(segmented_area.shape, dtype=bool))
            ISI2_mask_list_copy[np.ndarray.tolist(overlap_ratios).index(np.amax(overlap_ratios))] = \
                                                                         np.zeros(ISI2_mask_list_copy[0].shape)

    # reverse shift in centroid of each patch, used to identify patches
    translation_matrix = np.float32([[1,0,-y_shift],[0,1,-x_shift]])
    for nn in range(len(mask_list)):
        mask_list[nn] = cv2.warpAffine(mask_list[nn].astype(np.float), translation_matrix, \
                                                      mask_list[nn].shape).astype(np.bool)
    
    # add ordered list of masks to ISI2_patch_mask_list
    ISI2_patch_mask_list.append(mask_list)
    
    # plot results
    fig = plt.figure(figsize=(12,12))
    fig.suptitle((ISI2_list[ii]), fontsize=12)
    ax1 = fig.add_subplot(221)
    ax1.imshow(ISI2_sign_map_stack[ii], interpolation='nearest', cmap='jet')
    ax2 = fig.add_subplot(222)
    for jj in range(len(ISI2_mask_list)):
        segmented_mask = np.zeros(ISI2_mask_list[jj].shape)
        segmented_mask[:] = np.nan
        segmented_mask[ISI2_mask_list[jj] == True] = 1
        if ISI2_field_sign_list[jj] == -1:
            segmented_mask = segmented_mask * -1
        ax2.imshow(segmented_mask, interpolation='none', alpha=0.5, clim=(-1,1), cmap='jet')
    
    for jj in range(len(mean_field_sign_list)):
        segmented_mask = np.empty(ISI2_sign_map_stack[ii,:,:].shape)
        segmented_mask[:] = np.nan
        segmented_mask[mask_list[jj] == 1] = mean_field_sign_list[jj]
        if np.sum(mask_list[jj]) > 0:
            ax2.imshow(segmented_mask, interpolation='none', clim=(-1,1), cmap='jet')
            ax2.text(scipy.ndimage.measurements.center_of_mass(np.asarray(mask_list)[jj])[1], 
                     scipy.ndimage.measurements.center_of_mass(np.asarray(mask_list)[jj])[0], 
                     mean_area_name_list[jj], fontsize=10, color='white')    
    plt.show()
```

## 3.4 Count instances of each patch¶

In [23]:

```
ISI1_patch_count = np.zeros(len(mean_area_name_list), dtype=np.int)
ISI2_patch_count = np.zeros(len(mean_area_name_list), dtype=np.int)
ISI_patch_count = np.zeros(len(mean_area_name_list), dtype=np.int)

for kk in range(len(mean_area_name_list)):
    for ii in range(len(ISI1_list)):
        if np.sum(ISI1_patch_mask_list[ii][kk]) > 0:
            ISI1_patch_count[kk] = ISI1_patch_count[kk] + 1
        if np.sum(ISI2_patch_mask_list[ii][kk]) > 0:
            ISI2_patch_count[kk] = ISI2_patch_count[kk] + 1
        if np.sum(ISI1_patch_mask_list[ii][kk]) > 0 and np.sum(ISI2_patch_mask_list[ii][kk]) > 0:
            ISI_patch_count[kk] = ISI_patch_count[kk] + 1

for ii in range(len(mean_area_name_list)):
    print mean_area_name_list[ii], ISI1_patch_count[ii], ISI2_patch_count[ii], ISI_patch_count[ii]
```

```
V1 60 60 60
RL 60 60 60
LM 59 57 56
AM 59 53 52
PM 60 60 60
P 37 35 30
RLL 52 40 38
AL 58 57 56
LLA 35 28 18
MMA 54 48 42
MMP 58 55 54
```

# 4. Is there biological variation?¶

## Comparison of variability across mice and across imaging sessions¶

## 4.1 Find centroids of patches¶

In [24]:

```
ISI1_centroid_array_x = np.zeros((len(ISI1_list),len(mean_area_name_list)))
ISI1_centroid_array_y = np.zeros((len(ISI1_list),len(mean_area_name_list)))
ISI2_centroid_array_x = np.zeros((len(ISI1_list),len(mean_area_name_list)))
ISI2_centroid_array_y = np.zeros((len(ISI1_list),len(mean_area_name_list)))

for ii in range(len(ISI1_list)):
    for jj in range(len(mean_area_name_list)):
        ISI1_centroid_array_x[ii,jj] = scipy.ndimage.measurements.center_of_mass(ISI1_patch_mask_list[ii][jj])[0]
        ISI1_centroid_array_y[ii,jj] = scipy.ndimage.measurements.center_of_mass(ISI1_patch_mask_list[ii][jj])[1]
        ISI2_centroid_array_x[ii,jj] = scipy.ndimage.measurements.center_of_mass(ISI2_patch_mask_list[ii][jj])[0]
        ISI2_centroid_array_y[ii,jj] = scipy.ndimage.measurements.center_of_mass(ISI2_patch_mask_list[ii][jj])[1]

ISI1_centroid_array_x[ISI1_centroid_array_x == 0] = np.nan
ISI1_centroid_array_y[ISI1_centroid_array_y == 0] = np.nan
ISI2_centroid_array_x[ISI2_centroid_array_x == 0] = np.nan
ISI2_centroid_array_y[ISI2_centroid_array_y == 0] = np.nan

# x dimension is a-p axis with higher numbers corresonding to more posterior locations
# y dimension is m-l axis with higher numbers corresonding to more medial locations
```

```
C:\anaconda2\lib\site-packages\scipy\ndimage\measurements.py:1301: RuntimeWarning: invalid value encountered in double_scalars
  for dir in range(input.ndim)]
```

In [25]:

```
# plot centroids
display = np.full(signmap.shape, np.nan)
for ii in range(len(ISI1_list)):
    for jj in range(len(mean_field_sign_list)):
        if np.isfinite(ISI1_centroid_array_x[ii,jj]) and np.isfinite(ISI1_centroid_array_y[ii,jj]):
            display[np.int(ISI1_centroid_array_x[ii,jj])-1:np.int(ISI1_centroid_array_x[ii,jj]+1), \
                    np.int(ISI1_centroid_array_y[ii,jj])-1:np.int(ISI1_centroid_array_y[ii,jj])+1] = mean_field_sign_list[jj]
        if np.isfinite(ISI2_centroid_array_x[ii,jj]) and np.isfinite(ISI2_centroid_array_y[ii,jj]):
            display[np.int(ISI2_centroid_array_x[ii,jj])-1:np.int(ISI2_centroid_array_x[ii,jj]+1), \
                    np.int(ISI2_centroid_array_y[ii,jj])-1:np.int(ISI2_centroid_array_y[ii,jj])+1] = mean_field_sign_list[jj]

fig = plt.figure(figsize=(7,7))
ax1 = fig.add_subplot(111)
ax1.imshow(display, cmap='bwr')
plt.show()
```

## 4.2 Pairwise comparison of variability across mice and across measurements¶

### Paired patch distance (ppd) is the sum (across patches) of squares of centroid differences.¶

### To correct for missing patches, for each map, sum is divided by the number of patches.¶

In [26]:

```
# calculate distribution of ISI2-ISI1 differences
diff_betw_measurements = []
for ii in range(len(ISI1_list)):
    patch_count = 0
    centroid_cumulative_sum = 0
    for jj in range(len(mean_area_name_list)):
        if np.sum(ISI1_patch_mask_list[ii][jj]) > 0 and np.sum(ISI2_patch_mask_list[ii][jj]) > 0:
            centroid_cumulative_sum = centroid_cumulative_sum + \
                (ISI1_centroid_array_x[ii,jj] - ISI2_centroid_array_x[ii,jj])**2 + \
                (ISI1_centroid_array_y[ii,jj] - ISI2_centroid_array_y[ii,jj])**2 
            patch_count = patch_count + 1
    diff_betw_measurements.append(np.sqrt(centroid_cumulative_sum / patch_count))
diff_betw_measurements = np.asarray(diff_betw_measurements)
diff_betw_measurements[diff_betw_measurements == 0] = np.nan  

# calculate the distribution of pairwise mouse-to-mouse differences
diff_betw_mice = []
for ii in range(len(ISI1_list)):
    for kk in range(ii+1, len(ISI1_list)):
        patch_count_ISI1 = 0
        patch_count_ISI2 = 0
        centroid_cumulative_sum_ISI1 = 0
        centroid_cumulative_sum_ISI2 = 0
        for jj in range(len(mean_area_name_list)):
            
            if np.isfinite(ISI1_centroid_array_x[ii,jj]) and np.isfinite(ISI1_centroid_array_x[kk,jj]):
                centroid_cumulative_sum_ISI1 = centroid_cumulative_sum_ISI1 + \
                    (ISI1_centroid_array_x[ii,jj] - ISI1_centroid_array_x[kk,jj])**2 + \
                    (ISI1_centroid_array_y[ii,jj] - ISI1_centroid_array_y[kk,jj])**2
                patch_count_ISI1 = patch_count_ISI1 + 1
            
            if np.isfinite(ISI2_centroid_array_x[ii,jj]) and np.isfinite(ISI2_centroid_array_x[kk,jj]):
                centroid_cumulative_sum_ISI2 = centroid_cumulative_sum_ISI2 + \
                    (ISI2_centroid_array_x[ii,jj] - ISI2_centroid_array_x[kk,jj])**2 + \
                    (ISI2_centroid_array_y[ii,jj] - ISI2_centroid_array_y[kk,jj])**2
                patch_count_ISI2 = patch_count_ISI2 + 1
                
        diff_betw_mice.append(np.sqrt(centroid_cumulative_sum_ISI1 / patch_count_ISI1))
        diff_betw_mice.append(np.sqrt(centroid_cumulative_sum_ISI2 / patch_count_ISI2))
diff_betw_mice = np.asarray(diff_betw_mice)

#stats
_, p_val = scipy.stats.mannwhitneyu(diff_betw_mice, diff_betw_measurements)
print 'p value', p_val

# plot
fig = plt.figure(figsize=(8,5))
ax1 = fig.add_subplot(111)

hist_array = diff_betw_mice
y, x, _ = ax1.hist(hist_array, bins=25, normed=True, alpha=0.5, range=(0,150))
param = scipy.stats.lognorm.fit(hist_array, loc=40)
x = np.linspace(0,150,150)
pdf_fit = scipy.stats.lognorm.pdf(x, param[0], loc=param[1], scale=param[2])
ax1.plot(x,pdf_fit,'b-', )

hist_array = diff_betw_measurements
y, x, _ = ax1.hist(hist_array, bins=25, normed=True, alpha=0.5)
param = scipy.stats.lognorm.fit(hist_array, loc=10)
pdf_fit = scipy.stats.lognorm.pdf(x, param[0], loc=param[1], scale=param[2])
ax1.plot(x,pdf_fit,'r-')
ax1.set_xlim(0,150)

plt.xlabel('ppd')
plt.ylabel('normalized probability')

plt.show()
```

```
p value 2.4074704987941435e-09
```

## Conclusion: there's mouse-to-mouse variability¶

### Variability could be in location (translation), size (scale), rotation or shape (relative positions of visual areas), or in any combination of these 4.¶

# 5. Is there biological variation in size?¶

### Look at size of the overall map of visual areas (5.2) and of surface areas of individual patches (5.3).¶

## 5.1 First test for and remove translation differences between maps.¶

### Center each map, moving the centroid of V1 to the origin.¶

In [27]:

```
# test for x centroid

# calculate distribution of ISI2-ISI1 differences
diff_betw_measurements = []
for ii in range(len(ISI1_list)):
    diff_betw_measurements.append(ISI1_centroid_array_x[ii,0] - ISI2_centroid_array_x[ii,0])
diff_betw_measurements = np.asarray(diff_betw_measurements)

# calculate the distribution of pairwise mouse-to-mouse differences
diff_betw_mice = []
for ii in range(len(ISI1_list)):
    for kk in range(ii+1, len(ISI1_list)):
        diff_betw_mice.append(ISI1_centroid_array_x[ii,0] - ISI1_centroid_array_x[kk,0])
        diff_betw_mice.append(ISI2_centroid_array_x[ii,0] - ISI2_centroid_array_x[kk,0])
diff_betw_mice = np.asarray(diff_betw_mice)

#stats
_, p_val = scipy.stats.mannwhitneyu(diff_betw_mice, diff_betw_measurements)
print 'p value', p_val
print np.nanmean(diff_betw_measurements), np.nanmin(diff_betw_measurements), np.nanmax(diff_betw_measurements)
print np.nanmean(diff_betw_mice), np.nanmin(diff_betw_mice), np.nanmax(diff_betw_mice)

# plot
fig = plt.figure(figsize=(8,5))
ax1 = fig.add_subplot(111)
fig.suptitle('differences in V1 x centroids')

hist_array = diff_betw_mice
y, x, _ = ax1.hist(hist_array, bins=25, normed=True, alpha=0.5, range=(-100,100))

hist_array = diff_betw_measurements
y, x, _ = ax1.hist(hist_array, bins=25, normed=True, alpha=0.5, range=(-100,100))
ax1.set_xlim(-100,100)

plt.xlabel('V1 centroid')
plt.ylabel('normalized probability')

plt.show()
```

```
p value 0.029957274811440244
3.599198517345125 -25.548123956361223 35.944061845381555
-1.119529345796786 -77.52899482326029 76.22973150902658
```

In [28]:

```
# y centroid

# calculate distribution of ISI2-ISI1 differences
diff_betw_measurements = []
for ii in range(len(ISI1_list)):
    diff_betw_measurements.append(ISI1_centroid_array_y[ii,0] - ISI2_centroid_array_y[ii,0])
diff_betw_measurements = np.asarray(diff_betw_measurements)

# calculate the distribution of pairwise mouse-to-mouse differences
diff_betw_mice = []
for ii in range(len(ISI1_list)):
    for kk in range(ii+1, len(ISI1_list)):
        diff_betw_mice.append(ISI1_centroid_array_y[ii,0] - ISI1_centroid_array_y[kk,0])
        diff_betw_mice.append(ISI2_centroid_array_y[ii,0] - ISI2_centroid_array_y[kk,0])
diff_betw_mice = np.asarray(diff_betw_mice)

#stats
_, p_val = scipy.stats.mannwhitneyu(diff_betw_mice, diff_betw_measurements)
print 'p value', p_val
print np.nanmean(diff_betw_measurements), np.nanmin(diff_betw_measurements), np.nanmax(diff_betw_measurements)
print np.nanmean(diff_betw_mice), np.nanmin(diff_betw_mice), np.nanmax(diff_betw_mice)

# plot
fig = plt.figure(figsize=(8,5))
ax1 = fig.add_subplot(111)
fig.suptitle('differences in V1 y centroids')

hist_array = diff_betw_mice
y, x, _ = ax1.hist(hist_array, bins=25, normed=True, alpha=0.5, range=(-100,100))

hist_array = diff_betw_measurements
y, x, _ = ax1.hist(hist_array, bins=25, normed=True, alpha=0.5, range=(-100,100))
ax1.set_xlim(-100,100)

plt.xlabel('V1 centroid')
plt.ylabel('normalized probability')

plt.show()
```

```
p value 0.28061876851948775
-2.246206961518382 -16.56347426280911 10.017223235551967
-2.9424622695771125 -60.09500363756342 62.17603763468168
```

In [29]:

```
for ii in range(len(ISI1_list)):
    ISI1_centroid_array_x[ii,:] = ISI1_centroid_array_x[ii,:] - ISI1_centroid_array_x[ii,0]
    ISI1_centroid_array_y[ii,:] = ISI1_centroid_array_y[ii,:] - ISI1_centroid_array_y[ii,0]
    
    ISI2_centroid_array_x[ii,:] = ISI2_centroid_array_x[ii,:] - ISI2_centroid_array_x[ii,0]
    ISI2_centroid_array_y[ii,:] = ISI2_centroid_array_y[ii,:] - ISI2_centroid_array_y[ii,0]
```

In [30]:

```
# plot centroids
display = np.full(signmap.shape, np.nan)
for ii in range(len(ISI1_list)):
    for jj in range(len(mean_field_sign_list)):
        if np.isfinite(ISI1_centroid_array_x[ii,jj]) and np.isfinite(ISI1_centroid_array_y[ii,jj]):
            display[np.int(ISI1_centroid_array_x[ii,jj])+298:np.int(ISI1_centroid_array_x[ii,jj]+302), \
                    np.int(ISI1_centroid_array_y[ii,jj])+298:np.int(ISI1_centroid_array_y[ii,jj])+302] = mean_field_sign_list[jj]
        if np.isfinite(ISI2_centroid_array_x[ii,jj]) and np.isfinite(ISI2_centroid_array_y[ii,jj]):
            display[np.int(ISI2_centroid_array_x[ii,jj])+298:np.int(ISI2_centroid_array_x[ii,jj]+302), \
                    np.int(ISI2_centroid_array_y[ii,jj])+298:np.int(ISI2_centroid_array_y[ii,jj])+302] = mean_field_sign_list[jj]

fig = plt.figure(figsize=(7,7))
ax1 = fig.add_subplot(111)
ax1.imshow(display, cmap='bwr')
ax1.axhline(300, color='k', linewidth=1)
ax1.axvline(300, color='k', linewidth=1)
ax1.tick_params(
    axis='x',          # changes apply to the x-axis
    which='both',      # both major and minor ticks are affected
    bottom='off',      # ticks along the bottom edge are off
    labelbottom='off') # labels along the bottom edge are off
ax1.tick_params(
    axis='y',          # changes apply to the x-axis
    which='both',      # both major and minor ticks are affected
    left='off',      # ticks along the bottom edge are off
    labelleft='off') # labels along the bottom edge are off
plt.show()
```

## 5.2 Scale of overall map¶

### Calculate and compare centroid sizes (across mice vs across sessions).¶

### Centroid size is square root of the summed squared distances of each landmark from the centroid (page 60).¶

### Calculating centroid size from three landmarks: V1, RL and PM (since all maps contain these three patches).¶

In [31]:

```
# centroid arrays are in pixels so units of centroid size will be pixels.
# In mean_area_name_list, V1, RL and PM are entries 0, 1 and 4, respectively.

centroid_size_array = np.zeros((60,2))

for ii in range(len(ISI1_list)):
    # first find the centroid of the triangle
    x_ISI1_centroid = (ISI1_centroid_array_x[ii,0] + ISI1_centroid_array_x[ii,1] + ISI1_centroid_array_x[ii,4]) / 3
    y_ISI1_centroid = (ISI1_centroid_array_y[ii,0] + ISI1_centroid_array_y[ii,1] + ISI1_centroid_array_y[ii,4]) / 3
    
    # then calculate the sum of squared distances from the centroid
    centroid_size_ISI1 = ((ISI1_centroid_array_x[ii,0] - x_ISI1_centroid)**2 + \
                          (ISI1_centroid_array_x[ii,1] - x_ISI1_centroid)**2 + \
                          (ISI1_centroid_array_x[ii,4] - x_ISI1_centroid)**2 + \
                          (ISI1_centroid_array_y[ii,0] - y_ISI1_centroid)**2 + \
                          (ISI1_centroid_array_y[ii,1] - y_ISI1_centroid)**2 + \
                          (ISI1_centroid_array_y[ii,4] - y_ISI1_centroid)**2)**0.5
    
    centroid_size_array[ii,0] = centroid_size_ISI1

    # first find the centroid of the triangle
    x_ISI2_centroid = (ISI2_centroid_array_x[ii,0] + ISI2_centroid_array_x[ii,1] + ISI2_centroid_array_x[ii,4]) / 3
    y_ISI2_centroid = (ISI2_centroid_array_y[ii,0] + ISI2_centroid_array_y[ii,1] + ISI2_centroid_array_y[ii,4]) / 3

    # then calculate the sum of squared distances from the centroid
    centroid_size_ISI2 = ((ISI2_centroid_array_x[ii,0] - x_ISI2_centroid)**2 + \
                          (ISI2_centroid_array_x[ii,1] - x_ISI2_centroid)**2 + \
                          (ISI2_centroid_array_x[ii,4] - x_ISI2_centroid)**2 + \
                          (ISI2_centroid_array_y[ii,0] - y_ISI2_centroid)**2 + \
                          (ISI2_centroid_array_y[ii,1] - y_ISI2_centroid)**2 + \
                          (ISI2_centroid_array_y[ii,4] - y_ISI2_centroid)**2)**0.5
    
    centroid_size_array[ii,1] = centroid_size_ISI2
```

In [32]:

```
# compare mouse-to-mouse and between-session distributions of centroid size differences

# calculate distribution of ISI2-ISI1 differences
diff_betw_measurements = []
for ii in range(len(ISI1_list)):
    diff_betw_measurements.append(centroid_size_array[ii,0] - centroid_size_array[ii,1])
diff_betw_measurements = np.asarray(diff_betw_measurements)

# calculate the distribution of mouse-to-mouse differences
diff_betw_mice = []
for ii in range(len(centroid_size_array)):
    for jj in range(ii + 1, len(centroid_size_array)):
        diff_betw_mice.append(centroid_size_array[ii,0] - centroid_size_array[jj,0])
        diff_betw_mice.append(centroid_size_array[ii,1] - centroid_size_array[jj,1])
diff_betw_mice = np.asarray(diff_betw_mice)

# print stats
z_stat, p_val = scipy.stats.levene(diff_betw_mice, diff_betw_measurements)
print 'P = ', p_val

# plot
fig = plt.figure(figsize=(7,5))
ax1 = fig.add_subplot(111)

y, x, _ = ax1.hist(diff_betw_mice, bins=100, range=(-100,100), normed=True, alpha=0.5)
mu, sigma = scipy.stats.norm.fit(diff_betw_mice)
y = mlab.normpdf(x, mu, sigma)
ax1.plot(x, y, 'b-', linewidth=1)

y, x, _ = ax1.hist(diff_betw_measurements, bins=100, range=(-100,100), normed=True, alpha=0.5)
mu, sigma = scipy.stats.norm.fit(diff_betw_measurements)
y = mlab.normpdf(x, mu, sigma)
ax1.plot(x, y, 'r-', linewidth=1)

plt.xlabel('centroid size')
plt.ylabel('normalized probability')

ax1.set_xlim(-50,50)
plt.show()
```

```
P =  0.002557797462835884
```

## 5.3 Surface areas of individual patches¶

In [33]:

```
ISI1_surface_area_array = np.zeros((len(ISI1_patch_mask_list),len(ISI1_patch_mask_list[0])))
ISI2_surface_area_array = np.zeros((len(ISI1_patch_mask_list),len(ISI1_patch_mask_list[0])))

for ii in range(len(ISI1_patch_mask_list)):
    for jj in range(len(ISI1_patch_mask_list[0])):
        ISI1_surface_area_array[ii,jj] = np.sum(ISI1_patch_mask_list[ii][jj])
        ISI2_surface_area_array[ii,jj] = np.sum(ISI2_patch_mask_list[ii][jj])
        
# convert from pixels to mm2
ISI1_surface_area_array = ISI1_surface_area_array / 10000.
ISI2_surface_area_array = ISI2_surface_area_array / 10000.

ISI1_surface_area_array[ISI1_surface_area_array == 0] = np.nan
ISI2_surface_area_array[ISI2_surface_area_array == 0] = np.nan
```

In [34]:

```
#    ax2.plot(x, y, 'r-', linewidth=1, label='fdafsaf')
#    ax2.legend()
```

In [35]:

```
# calculate distributions of surface area differences
SA_stdev_list = []

for kk in range(ISI1_surface_area_array.shape[1]):
    # calculate the distribution of pairwise mouse-to-mouse differences
    diff_betw_mice = []
    for ii in range(len(ISI1_surface_area_array)):
        for jj in range(ii + 1, len(ISI1_surface_area_array)):
                if ISI1_surface_area_array[ii,kk] > 0 and ISI1_surface_area_array[jj,kk] > 0:
                    diff_betw_mice.append(ISI1_surface_area_array[ii,kk] - ISI1_surface_area_array[jj,kk])
                if ISI2_surface_area_array[ii,kk] > 0 and ISI2_surface_area_array[jj,kk] > 0:   
                    diff_betw_mice.append(ISI2_surface_area_array[ii,kk] - ISI2_surface_area_array[jj,kk])
    diff_betw_mice = np.asarray(diff_betw_mice)
    diff_betw_mice = diff_betw_mice - np.mean(diff_betw_mice)

    # calculate distribution of ISI2-ISI1 differences
    diff_betw_measurements = []
    for ii in range(len(ISI1_surface_area_array)):
        if ISI1_surface_area_array[ii,kk] > 0 and ISI2_surface_area_array[ii,kk] > 0:
            diff_betw_measurements.append(ISI1_surface_area_array[ii,kk] - ISI2_surface_area_array[ii,kk])
    diff_betw_measurements = np.asarray(diff_betw_measurements)
    diff_betw_measurements = diff_betw_measurements - np.mean(diff_betw_measurements)
    
    if np.var(diff_betw_mice) > np.var(diff_betw_measurements):
        SA_stdev_list.append((np.var(diff_betw_mice) - np.var(diff_betw_measurements))**0.5)
    else:
        SA_stdev_list.append(0)   


    # print stats
    z_stat, p_val = scipy.stats.levene(diff_betw_mice, diff_betw_measurements)
    
    print '________________________'
    print ' '
    print mean_area_name_list[kk], 'p =', p_val,
    if p_val < p_criterion / 11: # div by 11 for Bonferroni correction
        print 'p <', p_criterion / 11
    else:
        print 'no significant difference'
        
        
    # plots
    fig = plt.figure(figsize=(15,10))
    fig.suptitle((mean_area_name_list[kk], ISI1_patch_count[kk], ISI2_patch_count[kk]), fontsize=12)
    
    ax1 = fig.add_subplot(331)
    ax2 = fig.add_subplot(332)
    ax3 = fig.add_subplot(333)
    
    y, x, _ = ax1.hist(diff_betw_mice, bins=100, range=(-3,3), normed=True, alpha=0.5)
    mu, sigma = scipy.stats.norm.fit(diff_betw_mice[~np.isnan(diff_betw_mice)])
    y = mlab.normpdf(x, mu, sigma)
    sigma1 = sigma
    
    ax1.plot(x, y, 'b-', linewidth=1)
    y, x, _ = ax1.hist(diff_betw_measurements, bins=100, range=(-3,3), normed=True, alpha=0.5)
    mu, sigma = scipy.stats.norm.fit(diff_betw_measurements[~np.isnan(diff_betw_measurements)])
    y = mlab.normpdf(x, mu, sigma)
    sigma2 = sigma
    ax1.plot(x, y, 'r-', linewidth=1)
    
    y, x, _ = ax2.hist(diff_betw_mice, bins=100, range=(-np.int(np.ceil(np.amax(diff_betw_mice)/40)),
                                        np.int(np.ceil(np.amax(diff_betw_mice)/40))), normed=True, alpha=0.5)
    mu, sigma = scipy.stats.norm.fit(diff_betw_mice[~np.isnan(diff_betw_mice)])
    y = mlab.normpdf(x, mu, sigma)
    ax2.plot(x, y, 'b-', linewidth=1) 
    ax3.plot(x, y/np.amax(y), 'b-', linewidth=1)
    y, x, _ = ax2.hist(diff_betw_measurements, bins=100, range=(-np.int(np.ceil(np.amax(diff_betw_mice)/40)),
                                        np.int(np.ceil(np.amax(diff_betw_mice)/40))), normed=True, alpha=0.5)
    mu, sigma = scipy.stats.norm.fit(diff_betw_measurements[~np.isnan(diff_betw_measurements)])
    y = mlab.normpdf(x, mu, sigma)
    ax2.plot(x, y, 'r-', linewidth=1)
    ax3.plot(x, y/np.amax(y), 'r-', linewidth=1)  
    
    print 'mouse-to-mouse std', sigma1
    print 'measurement std', sigma2
    
    plt.show()
```

```
________________________
 
V1 p = 1.1257414196411455e-05 p < 0.00454545454545
mouse-to-mouse std 0.5584817550912341
measurement std 0.3264736560071373
```

```
________________________
 
RL p = 0.06816790473444982 no significant difference
mouse-to-mouse std 0.7282245916566408
measurement std 0.6002401303066596
```

```
________________________
 
LM p = 0.029600987878776533 no significant difference
mouse-to-mouse std 0.32378733088767614
measurement std 0.2816110932551979
```

```
________________________
 
AM p = 0.009322040773199092 no significant difference
mouse-to-mouse std 0.17772082795195784
measurement std 0.12079655097340736
```

```
________________________
 
PM p = 0.0656185038018344 no significant difference
mouse-to-mouse std 0.5616408642903413
measurement std 0.46510150221633223
```

```
________________________
 
P p = 0.09463923154716164 no significant difference
mouse-to-mouse std 0.5578407434388895
measurement std 0.423452979942546
```

```
________________________
 
RLL p = 0.7774065720800564 no significant difference
mouse-to-mouse std 0.3464390656322104
measurement std 0.34542908670296313
```

```
________________________
 
AL p = 0.012979733338286973 no significant difference
mouse-to-mouse std 0.16788799114442876
measurement std 0.12115368465763823
```

```
________________________
 
LLA p = 0.08507193363371389 no significant difference
mouse-to-mouse std 0.18968570695732057
measurement std 0.14984591396530894
```

```
________________________
 
MMA p = 0.6155384825860061 no significant difference
mouse-to-mouse std 0.6255334648274241
measurement std 0.7116152558857188
```

```
________________________
 
MMP p = 0.8431576939433121 no significant difference
mouse-to-mouse std 0.44672186931856017
measurement std 0.46744698478035657
```

Note on statistics:
To compare population variances (two populations with equal means), have 3 approaches:
F-test
Bartlett
Levene
Code:
# two way F-test
p\_val = scipy.stats.f.cdf(np.var(dist1) / np.var(dist2),
len(dist1)-1, len(dist2)-1)
print 'F =', np.var(dist1) / np.var(dist2), 'p =', p\_val
# one way F-test
z\_stat, p\_val = scipy.stats.f\_oneway(dist1,dist2)
print 'F =', z\_stat, 'p =', p\_val
# Bartlett
z\_stat, p\_val = scipy.stats.bartlett(dist1,dist2)
print 'B =', z\_stat, 'p =', p\_val
# Levene
z\_stat, p\_val = scipy.stats.levene(dist1,dist2)
print 'L =', z\_stat, 'p =', p\_val
From the literaure,
F-test is sensitive to different sample sizes.
Bartlett is sensitive to departures from normal distributions.
Levene is relatively insensitive to different sample sizes, but sensitive to some departures from normalty, particularly distribution assymetry. Assymetry typically elevates type I errors (i.e. increases the probability of a false positive). In our data set, departure from normality is likely greater than asymmetry so the most robest test (in terms of avoiding type I errors) will be Levene.
In some situations when comparing 2 samples (as we are here and including the comparison of two normal distributions), Levene is prone to type II errors (i.e. false negatives). Hence the greatest risk with Levene is probably that we will conclude there is no difference when the distributions are different.
In practice,
on surface\_area distributions:
two-way F test gives p-value of ~1 for all patches.
one-way F test gives p-value = 1 for all patches.
Bartlett gives values that appear to bear little relation to the similarity of the Gaussian distributions (fits).
Levene gives p-values consistent with apparent similarity of normal distributions (fits).

### 5.3.1 Check for correlated differences in patch size¶

In [36]:

```
# first, average ISI1 and ISI1 sizes
norm_area_array = np.zeros((2,60,11))
norm_area_array[0] = ISI1_surface_area_array
norm_area_array[1] = ISI2_surface_area_array
norm_area_array = np.nanmean(norm_area_array, axis=0)

# calc correlation
spearman_arrays = scipy.stats.spearmanr(norm_area_array, axis=0)

# plot correlation coefficients
plt.imshow(spearman_arrays[0], cmap='bwr', clim=(-1,1))
plt.xticks(range(11), mean_area_name_list)
plt.yticks(range(11), mean_area_name_list)
plt.colorbar()
plt.show()
```

```
C:\anaconda2\lib\site-packages\ipykernel_launcher.py:5: RuntimeWarning: Mean of empty slice
  """
```

In [37]:

```
# plot p-values
plt.imshow(spearman_arrays[1], cmap='bwr', clim=(0,1))
plt.xticks(range(11), mean_area_name_list)
plt.yticks(range(11), mean_area_name_list)
plt.colorbar()
plt.show()
```

In [38]:

```
# plot significant p-values
pval_array = np.copy(spearman_arrays[1])
pval_array[pval_array > 0.05/11] = np.nan

plt.imshow(pval_array, cmap='bwr', clim=(0,1))
plt.xticks(range(11), mean_area_name_list)
plt.yticks(range(11), mean_area_name_list)
plt.colorbar()
plt.show()
```

# 6. Is there biological variation in the shape of the overall map?¶

## Eliminate differences scale and rotation and determine whether biological variation is lost.¶

## 6.1 Eliminate effects of scale¶

### Scale by centroid size (calculated above).¶

In [39]:

```
# normalize by centroid size
for ii in range(len(ISI1_list)):
    ISI1_centroid_array_x[ii,:] = (ISI1_centroid_array_x[ii,:] / centroid_size_array[ii,0]) * np.mean(centroid_size_array)
    ISI1_centroid_array_y[ii,:] = (ISI1_centroid_array_y[ii,:] / centroid_size_array[ii,0]) * np.mean(centroid_size_array)
    ISI2_centroid_array_x[ii,:] = (ISI2_centroid_array_x[ii,:] / centroid_size_array[ii,1]) * np.mean(centroid_size_array)
    ISI2_centroid_array_y[ii,:] = (ISI2_centroid_array_y[ii,:] / centroid_size_array[ii,1]) * np.mean(centroid_size_array)
```

In [40]:

```
# plot centroids
display = np.full(signmap.shape, np.nan)
for ii in range(len(ISI1_list)):
    for jj in range(len(mean_field_sign_list)):
        if np.isfinite(ISI1_centroid_array_x[ii,jj]) and np.isfinite(ISI1_centroid_array_y[ii,jj]):
            display[np.int(ISI1_centroid_array_x[ii,jj])+298:np.int(ISI1_centroid_array_x[ii,jj]+302), \
                    np.int(ISI1_centroid_array_y[ii,jj])+298:np.int(ISI1_centroid_array_y[ii,jj])+302] = mean_field_sign_list[jj]
        if np.isfinite(ISI2_centroid_array_x[ii,jj]) and np.isfinite(ISI2_centroid_array_y[ii,jj]):
            display[np.int(ISI2_centroid_array_x[ii,jj])+298:np.int(ISI2_centroid_array_x[ii,jj]+302), \
                    np.int(ISI2_centroid_array_y[ii,jj])+298:np.int(ISI2_centroid_array_y[ii,jj])+302] = mean_field_sign_list[jj]

fig = plt.figure(figsize=(7,7))
ax1 = fig.add_subplot(111)
ax1.imshow(display, cmap='bwr')
ax1.axhline(300, color='k', linewidth=1)
ax1.axvline(300, color='k', linewidth=1)
ax1.tick_params(
    axis='x',          # changes apply to the x-axis
    which='both',      # both major and minor ticks are affected
    bottom='off',      # ticks along the bottom edge are off
    labelbottom='off') # labels along the bottom edge are off
ax1.tick_params(
    axis='y',          # changes apply to the x-axis
    which='both',      # both major and minor ticks are affected
    left='off',      # ticks along the bottom edge are off
    labelleft='off') # labels along the bottom edge are off
plt.show()
```

## 6.2 Eliminate effects of rotation: rotate each map about the origin¶

In [41]:

```
# plot centroids
display = np.full(signmap.shape, np.nan)
for ii in range(len(ISI1_list)):
    for jj in range(len(mean_field_sign_list)):
        if np.isfinite(ISI1_centroid_array_x[ii,jj]) and np.isfinite(ISI1_centroid_array_y[ii,jj]):
            display[np.int(ISI1_centroid_array_x[ii,jj])+298:np.int(ISI1_centroid_array_x[ii,jj]+302), \
                    np.int(ISI1_centroid_array_y[ii,jj])+298:np.int(ISI1_centroid_array_y[ii,jj])+302] = mean_field_sign_list[jj]
        if np.isfinite(ISI2_centroid_array_x[ii,jj]) and np.isfinite(ISI2_centroid_array_y[ii,jj]):
            display[np.int(ISI2_centroid_array_x[ii,jj])+298:np.int(ISI2_centroid_array_x[ii,jj]+302), \
                    np.int(ISI2_centroid_array_y[ii,jj])+298:np.int(ISI2_centroid_array_y[ii,jj])+302] = mean_field_sign_list[jj]

fig = plt.figure(figsize=(7,7))
ax1 = fig.add_subplot(111)
ax1.imshow(display, cmap='bwr')
ax1.axhline(300, color='k', linewidth=1)
ax1.axvline(300, color='k', linewidth=1)
ax1.tick_params(
    axis='x',          # changes apply to the x-axis
    which='both',      # both major and minor ticks are affected
    bottom='off',      # ticks along the bottom edge are off
    labelbottom='off') # labels along the bottom edge are off
ax1.tick_params(
    axis='y',          # changes apply to the x-axis
    which='both',      # both major and minor ticks are affected
    left='off',      # ticks along the bottom edge are off
    labelleft='off') # labels along the bottom edge are off
plt.show()
```

In [42]:

```
# rotate all to 1st ISI1 map
angle_array = np.zeros((len(ISI1_list),2))
for ii in range(len(ISI1_list)):
    # ISI1, find angle
    Xr = ISI1_centroid_array_x[0,:]
    Yr = ISI1_centroid_array_y[0,:]
    
    Xt = ISI1_centroid_array_x[ii,:]
    Yt = ISI1_centroid_array_y[ii,:]
    
    angle = np.arctan(np.nansum(Yr*Xt - Xr*Yt)/np.nansum(Xr*Xt + Yr*Yt))
    rotation_matrix = np.array([[np.cos((angle/180.) * np.pi), -np.sin((angle/180.) * np.pi)],
                                [np.sin((angle/180.) * np.pi),  np.cos((angle/180.) * np.pi)]])
    angle_array[ii,0] = angle
    
    # ISI2, find angle
    Xr = ISI1_centroid_array_x[0,:]
    Yr = ISI1_centroid_array_y[0,:]
    
    Xt = ISI2_centroid_array_x[ii,:]
    Yt = ISI2_centroid_array_y[ii,:]
    
    angle = np.arctan(np.nansum(Yr*Xt - Xr*Yt)/np.nansum(Xr*Xt + Yr*Yt))
    rotation_matrix = np.array([[np.cos((angle/180.) * np.pi), -np.sin((angle/180.) * np.pi)],
                                [np.sin((angle/180.) * np.pi),  np.cos((angle/180.) * np.pi)]])
    angle_array[ii,1] = angle
```

In [43]:

```
# Are the mouse-to-mouse and session-to-session differences in rotation different?

# calculate distribution of ISI2-ISI1 differences
diff_betw_measurements = []
for ii in range(len(ISI1_list)):
    diff_betw_measurements.append(angle_array[ii,0] - angle_array[ii,1])
diff_betw_measurements = np.asarray(diff_betw_measurements)

# calculate the distribution of pairwise mouse-to-mouse differences
diff_betw_mice = []
for ii in range(len(ISI1_list)):
    for kk in range(ii+1, len(ISI1_list)):
        diff_betw_mice.append(angle_array[ii,0] - angle_array[kk,0])
        diff_betw_mice.append(angle_array[ii,1] - angle_array[kk,1])
diff_betw_mice = np.asarray(diff_betw_mice)

#stats
_, p_val = scipy.stats.mannwhitneyu(diff_betw_mice, diff_betw_measurements)
print 'p value', p_val

# plot
fig = plt.figure(figsize=(8,5))
ax1 = fig.add_subplot(111)
fig.suptitle('differences in rotation')

hist_array = diff_betw_mice
y, x, _ = ax1.hist(hist_array, bins=25, normed=True, alpha=0.5, range=(-1,1))

hist_array = diff_betw_measurements
y, x, _ = ax1.hist(hist_array, bins=25, normed=True, alpha=0.5, range=(-1,1))
ax1.set_xlim(-1,1)

plt.xlabel('rotation')
plt.ylabel('normalized probability')

plt.show()
```

```
p value 0.3074489134812076
```

## 6.3 Re-test for biological variation¶

In [44]:

```
# calculate distribution of ISI2-ISI1 differences
diff_betw_measurements = []
for ii in range(len(ISI1_list)):
    patch_count = 0
    centroid_cumulative_sum = 0
    for jj in range(len(mean_area_name_list)):
        if np.sum(ISI1_patch_mask_list[ii][jj]) > 0 and np.sum(ISI2_patch_mask_list[ii][jj]) > 0:
            centroid_cumulative_sum = centroid_cumulative_sum + \
                (ISI1_centroid_array_x[ii,jj] - ISI2_centroid_array_x[ii,jj])**2 + \
                (ISI1_centroid_array_y[ii,jj] - ISI2_centroid_array_y[ii,jj])**2 
            patch_count = patch_count + 1
    diff_betw_measurements.append(np.sqrt(centroid_cumulative_sum / patch_count))
diff_betw_measurements = np.asarray(diff_betw_measurements)
diff_betw_measurements[diff_betw_measurements == 0] = np.nan  

# calculate the distribution of pairwise mouse-to-mouse differences
diff_betw_mice = []
for ii in range(len(ISI1_list)):
    for kk in range(ii+1, len(ISI1_list)):
        patch_count_ISI1 = 0
        patch_count_ISI2 = 0
        centroid_cumulative_sum_ISI1 = 0
        centroid_cumulative_sum_ISI2 = 0
        for jj in range(len(mean_area_name_list)):

            if np.isfinite(ISI1_centroid_array_x[ii,jj]) and np.isfinite(ISI1_centroid_array_x[kk,jj]):
                centroid_cumulative_sum_ISI1 = centroid_cumulative_sum_ISI1 + \
                    (ISI1_centroid_array_x[ii,jj] - ISI1_centroid_array_x[kk,jj])**2 + \
                    (ISI1_centroid_array_y[ii,jj] - ISI1_centroid_array_y[kk,jj])**2
                patch_count_ISI1 = patch_count_ISI1 + 1
                        
            if np.isfinite(ISI2_centroid_array_x[ii,jj]) and np.isfinite(ISI2_centroid_array_x[kk,jj]):
                centroid_cumulative_sum_ISI2 = centroid_cumulative_sum_ISI2 + \
                    (ISI2_centroid_array_x[ii,jj] - ISI2_centroid_array_x[kk,jj])**2 + \
                    (ISI2_centroid_array_y[ii,jj] - ISI2_centroid_array_y[kk,jj])**2
                patch_count_ISI2 = patch_count_ISI2 + 1
                
        diff_betw_mice.append(np.sqrt(centroid_cumulative_sum_ISI1 / patch_count_ISI1))
        diff_betw_mice.append(np.sqrt(centroid_cumulative_sum_ISI2 / patch_count_ISI2))
diff_betw_mice = np.asarray(diff_betw_mice)

#stats
_, p_val = scipy.stats.mannwhitneyu(diff_betw_mice, diff_betw_measurements)
print 'p value', p_val

# plot
fig = plt.figure(figsize=(8,5))
ax1 = fig.add_subplot(111)

hist_array = diff_betw_mice
y, x, _ = ax1.hist(hist_array, bins=25, normed=True, alpha=0.5, range=(0,150))
param = scipy.stats.lognorm.fit(hist_array, loc=20)
x = np.linspace(0,150,150)
pdf_fit = scipy.stats.lognorm.pdf(x, param[0], loc=param[1], scale=param[2])
ax1.plot(x,pdf_fit,'b-')

hist_array = diff_betw_measurements
y, x, _ = ax1.hist(hist_array, bins=25, normed=True, alpha=0.5, range=(0,150))
param = scipy.stats.lognorm.fit(hist_array, loc=10)
x = np.linspace(0,150,150)
pdf_fit = scipy.stats.lognorm.pdf(x, param[0], loc=param[1], scale=param[2])
ax1.plot(x,pdf_fit,'r-')
ax1.set_xlim(0,150)

plt.xlabel('ppd')
plt.ylabel('normalized probability')

plt.show()
```

```
p value 0.0006182718424556575
```

## Conclusion: There's biological variation in shape.¶

## 6.5 Biological variation of centroid position for each patch¶

In [45]:

```
# x dimension is a-p axis with higher numbers corresonding to more posterior locations
# y dimension is m-l axis with higher numbers corresonding to more medial locations

# convert centroid arrays from pixels to um.
# * 10 since there pixels in CCF are 10 x 10 x 10 um.
ISI1_centroid_array_x = ISI1_centroid_array_x * 10
ISI1_centroid_array_y = ISI1_centroid_array_y * 10
ISI2_centroid_array_x = ISI2_centroid_array_x * 10
ISI2_centroid_array_y = ISI2_centroid_array_y * 10

stdev_array = np.zeros((11,4))

for kk in range(ISI1_centroid_array_x.shape[1]):
    # calculate the distribution of pairwise mouse-to-mouse differences
    diff_betw_mice_x = []
    for ii in range(len(ISI1_centroid_array_x)):
        for jj in range(ii + 1,len(ISI1_centroid_array_x)):
                if np.isfinite(ISI1_centroid_array_x[ii,kk])and np.isfinite(ISI1_centroid_array_x[jj,kk]):
                    diff_betw_mice_x.append(ISI1_centroid_array_x[ii,kk] - ISI1_centroid_array_x[jj,kk])
                if np.isfinite(ISI2_centroid_array_x[ii,kk])and np.isfinite(ISI2_centroid_array_x[jj,kk]):   
                    diff_betw_mice_x.append(ISI2_centroid_array_x[ii,kk] - ISI2_centroid_array_x[jj,kk])
    diff_betw_mice_x = np.asarray(diff_betw_mice_x)
    diff_betw_mice_x = diff_betw_mice_x - np.mean(diff_betw_mice_x)
    
    # calculate distribution of ISI2-ISI1 differences
    diff_betw_measurements_x = []
    for ii in range(len(ISI1_centroid_array_x)):
        if np.isfinite(ISI1_centroid_array_x[ii,kk]) and np.isfinite(ISI2_centroid_array_x[ii,kk]):
            diff_betw_measurements_x.append(ISI1_centroid_array_x[ii,kk] - ISI2_centroid_array_x[ii,kk])
    diff_betw_measurements_x = np.asarray(diff_betw_measurements_x)
    diff_betw_measurements_x = diff_betw_measurements_x - np.mean(diff_betw_measurements_x)
            
    # calculate the distribution of pairwise mouse-to-mouse differences
    diff_betw_mice_y = []
    for ii in range(len(ISI1_centroid_array_y)):
        for jj in range(ii + 1,len(ISI1_centroid_array_y)):
            if jj != ii:
                if np.isfinite(ISI1_centroid_array_y[ii,kk]) and np.isfinite(ISI1_centroid_array_y[jj,kk]):
                    diff_betw_mice_y.append(ISI1_centroid_array_y[ii,kk] - ISI1_centroid_array_y[jj,kk])
                if np.isfinite(ISI2_centroid_array_y[ii,kk]) and np.isfinite(ISI2_centroid_array_y[jj,kk]):
                    diff_betw_mice_y.append(ISI2_centroid_array_y[ii,kk] - ISI2_centroid_array_y[jj,kk])
    diff_betw_mice_y = np.asarray(diff_betw_mice_y)
    diff_betw_mice_y = diff_betw_mice_y - np.mean(diff_betw_mice_y)

    # calculate distribution of ISI2-ISI1 differences
    diff_betw_measurements_y = []
    for ii in range(len(ISI1_centroid_array_y)):
        if np.isfinite(ISI1_centroid_array_y[ii,kk]) and np.isfinite(ISI2_centroid_array_y[ii,kk]):
            diff_betw_measurements_y.append(ISI1_centroid_array_y[ii,kk] - ISI2_centroid_array_y[ii,kk])
    diff_betw_measurements_y = np.asarray(diff_betw_measurements_y)
    diff_betw_measurements_y = diff_betw_measurements_y - np.mean(diff_betw_measurements_y)
    
    
    # print stats
    print '________________________'
    print ' '
    print mean_area_name_list[kk]
    print ISI1_patch_count[kk], 'ISI1 maps and', ISI2_patch_count[kk], 'ISI2 maps'
    z_stat, p_val = scipy.stats.levene(diff_betw_mice_x, diff_betw_measurements_x)
    print 'x centroid', 'p =', p_val,
    if p_val < p_criterion / 11: # div by 11 for Bonferroni correction
        print 'p <', p_criterion / 11
    else:
        print 'no significant difference'
    z_stat, p_val = scipy.stats.levene(diff_betw_mice_y, diff_betw_measurements_y)
    print 'y centroid', 'p =', p_val,
    if p_val < p_criterion / 11: # div by 11 for Bonferroni correction
        print 'p <', p_criterion / 11
    else:
        print 'no significant difference'
    
    # plots
    fig = plt.figure(figsize=(15,10))
    ax1 = fig.add_subplot(421)
    ax2 = fig.add_subplot(422)
    
    y, x, _ = ax1.hist(diff_betw_mice_x, bins=range(-1500,1500,25), normed=True, alpha=0.5)
    mu, sigma = scipy.stats.norm.fit(diff_betw_mice_x[~np.isnan(diff_betw_mice_x)])
    print mu, sigma
    y = mlab.normpdf(x, mu, sigma)
    sigma1 = sigma
    ax1.plot(x, y, 'b-', linewidth=1)
    ax2.plot(x, y/np.amax(y), 'b-', linewidth=1)
    y, x, _ = ax1.hist(diff_betw_measurements_x, bins=range(-1500,1500,25), normed=True, alpha=0.5)
    mu, sigma = scipy.stats.norm.fit(diff_betw_measurements_x[~np.isnan(diff_betw_measurements_x)])
    print mu, sigma
    y = mlab.normpdf(x, mu, sigma)
    sigma2 = sigma
    ax1.plot(x, y, 'r-', linewidth=1)
    ax2.plot(x, y/np.amax(y), 'r-', linewidth=1)
    
    ax3 = fig.add_subplot(423)
    ax4 = fig.add_subplot(424)
    
    y, x, _ = ax3.hist(diff_betw_mice_y, bins=range(-1500,1500,25), normed=True, alpha=0.5)
    mu, sigma = scipy.stats.norm.fit(diff_betw_mice_y[~np.isnan(diff_betw_mice_y)])
    print mu, sigma
    y = mlab.normpdf(x, mu, sigma)
    sigma3 = sigma
    ax3.plot(x, y, 'b-', linewidth=1)
    ax4.plot(x, y/np.amax(y), 'b-', linewidth=1)
    y, x, _ = ax3.hist(diff_betw_measurements_y, bins=range(-1500,1500,25), normed=True, alpha=0.5)
    mu, sigma = scipy.stats.norm.fit(diff_betw_measurements_y[~np.isnan(diff_betw_measurements_y)])
    print mu, sigma
    sigma4 = sigma
    y = mlab.normpdf(x, mu, sigma)
    ax3.plot(x, y, 'r-', linewidth=1)
    ax4.plot(x, y/np.amax(y), 'r-', linewidth=1)
    
    # record stdev of each distribution
    stdev_array[kk,:] = [sigma1, sigma2, sigma3, sigma4]
    
    plt.show()
```

```
________________________
 
V1
60 ISI1 maps and 60 ISI2 maps
x centroid p = nan no significant difference
y centroid p = nan no significant difference
0.0 0.0
```

```
C:\anaconda2\lib\site-packages\scipy\stats\morestats.py:1965: RuntimeWarning: invalid value encountered in double_scalars
  W = numer / denom
C:\anaconda2\lib\site-packages\scipy\stats\_distn_infrastructure.py:879: RuntimeWarning: invalid value encountered in greater
  return (self.a < x) & (x < self.b)
C:\anaconda2\lib\site-packages\scipy\stats\_distn_infrastructure.py:879: RuntimeWarning: invalid value encountered in less
  return (self.a < x) & (x < self.b)
C:\anaconda2\lib\site-packages\scipy\stats\_distn_infrastructure.py:1821: RuntimeWarning: invalid value encountered in less_equal
  cond2 = cond0 & (x <= self.a)
C:\anaconda2\lib\site-packages\matplotlib\mlab.py:1544: RuntimeWarning: divide by zero encountered in double_scalars
  return 1./(np.sqrt(2*np.pi)*sigma)*np.exp(-0.5 * (1./sigma*(x - mu))**2)
C:\anaconda2\lib\site-packages\matplotlib\mlab.py:1544: RuntimeWarning: invalid value encountered in multiply
  return 1./(np.sqrt(2*np.pi)*sigma)*np.exp(-0.5 * (1./sigma*(x - mu))**2)
C:\anaconda2\lib\site-packages\numpy\core\_methods.py:26: RuntimeWarning: invalid value encountered in reduce
  return umr_maximum(a, axis, None, out, keepdims)
```

```
0.0 0.0
0.0 0.0
0.0 0.0
```

```
________________________
 
RL
60 ISI1 maps and 60 ISI2 maps
x centroid p = 0.22834274305913688 no significant difference
y centroid p = 0.21973966292596203 no significant difference
4.110710516487811e-15 299.78800132206175
-1.1842378929335004e-14 266.96677228799507
-3.596871701926835e-15 283.396422730116
-3.481659405224491e-14 263.7531504808972
```

```
________________________
 
LM
59 ISI1 maps and 57 ISI2 maps
x centroid p = 0.5758019912135728 no significant difference
y centroid p = 0.12086632844308258 no significant difference
-2.2001686163239873e-15 343.05402924180777
2.030122102171715e-14 349.8753765679428
1.1000843081619937e-14 237.94692918318216
1.4210854715202004e-14 222.3799194332037
```

```
________________________
 
AM
59 ISI1 maps and 53 ISI2 maps
x centroid p = 0.016617918283238612 no significant difference
y centroid p = 0.21977979453279822 no significant difference
-7.066323354661793e-15 304.34647066194407
1.1477998039201619e-14 244.28489092346098
-4.710882236441195e-15 350.1480885078326
-7.651998692801079e-15 326.95878483033914
```

```
________________________
 
PM
60 ISI1 maps and 60 ISI2 maps
x centroid p = 0.001429758881784137 p < 0.00454545454545
y centroid p = 0.003636866926706795 p < 0.00454545454545
-1.2332131549463433e-14 372.46456005090323
-4.736951571734001e-15 266.84602391704533
-2.0553552582439057e-15 181.34859880220841
0.0 136.82145696121032
```

```
________________________
 
P
37 ISI1 maps and 35 ISI2 maps
x centroid p = 0.4472359746162248 no significant difference
y centroid p = 0.02367764774295802 no significant difference
0.0 349.4923247694777
1.4210854715202005e-15 341.0724163835385
4.03899312444758e-14 804.0280645512291
-1.8000415972589204e-14 541.7473573426652
```

```
________________________
 
RLL
52 ISI1 maps and 40 ISI2 maps
x centroid p = 0.11355673949811017 no significant difference
y centroid p = 0.42975440862193204 no significant difference
-3.4548706620054256e-15 290.77007686157884
-1.271497527149653e-14 229.7184001442027
-5.182305993008138e-15 347.3171439955895
0.0 331.12656493841223
```

```
________________________
 
AL
58 ISI1 maps and 57 ISI2 maps
x centroid p = 0.002536336591880772 p < 0.00454545454545
y centroid p = 0.00607238917255242 no significant difference
-6.718335747168445e-15 214.75889158790116
2.5376526277146436e-15 161.37727082708548
8.957780996224593e-15 286.3488707038009
-1.9286159970631292e-14 205.23216120917192
```

```
________________________
 
LLA
35 ISI1 maps and 28 ISI2 maps
x centroid p = 0.04924948615542032 no significant difference
y centroid p = 0.2520686934618586 no significant difference
1.0282057265675447e-14 308.0963565213279
1.105288700071267e-14 238.8892345982065
-1.1216789744373215e-14 310.63089604457883
1.2631870857957336e-14 261.7027718819636
```

```
________________________
 
MMA
54 ISI1 maps and 48 ISI2 maps
x centroid p = 0.6216886647471349 no significant difference
y centroid p = 0.4958228415800213 no significant difference
0.0 349.4227958053162
1.3534147347811431e-15 337.84056807383587
1.421640799957684e-15 289.137693922279
1.0150610510858574e-14 296.5721599265782
```

```
________________________
 
MMP
58 ISI1 maps and 55 ISI2 maps
x centroid p = 0.5001877053170468 no significant difference
y centroid p = 0.3309895915393726 no significant difference
-4.637321615158334e-15 408.4248900630981
9.473903143468002e-15 364.7248727399641
-1.1593304037895834e-14 363.4871924058713
-1.394769073899456e-14 329.74856044285934
```

In [46]:

```
stdev_array
```

Out[46]:

```
array([[  0.        ,   0.        ,   0.        ,   0.        ],
       [299.78800132, 266.96677229, 283.39642273, 263.75315048],
       [343.05402924, 349.87537657, 237.94692918, 222.37991943],
       [304.34647066, 244.28489092, 350.14808851, 326.95878483],
       [372.46456005, 266.84602392, 181.3485988 , 136.82145696],
       [349.49232477, 341.07241638, 804.02806455, 541.74735734],
       [290.77007686, 229.71840014, 347.317144  , 331.12656494],
       [214.75889159, 161.37727083, 286.3488707 , 205.23216121],
       [308.09635652, 238.8892346 , 310.63089604, 261.70277188],
       [349.42279581, 337.84056807, 289.13769392, 296.57215993],
       [408.42489006, 364.72487274, 363.48719241, 329.74856044]])
```

In [47]:

```
# stdev for centroid of each patch
biol_stdev_array = np.zeros((11,2))

for ii in range(len(mean_area_name_list)):
    biol_stdev_array[ii,0] = (stdev_array[ii,0]**2 - stdev_array[ii,1]**2)**0.5
    biol_stdev_array[ii,1] = (stdev_array[ii,2]**2 - stdev_array[ii,3]**2)**0.5
biol_stdev_array[np.isnan(biol_stdev_array) == 1] = 0

print 'x and y stdev (in um) for patches', mean_area_name_list
print biol_stdev_array
```

```
x and y stdev (in um) for patches ['V1', 'RL', 'LM', 'AM', 'PM', 'P', 'RLL', 'AL', 'LLA', 'MMA', 'MMP']
[[  0.           0.        ]
 [136.38763958 103.6716356 ]
 [  0.          84.65171316]
 [181.52593829 125.30617267]
 [259.85197327 119.02606102]
 [ 76.25281539 594.11356608]
 [178.2601869  104.80647168]
 [141.69953414 199.68834658]
 [194.56438136 167.34160501]
 [ 89.2190607    0.        ]
 [183.81147415 152.93405745]]
```

```
C:\anaconda2\lib\site-packages\ipykernel_launcher.py:5: RuntimeWarning: invalid value encountered in double_scalars
  """
C:\anaconda2\lib\site-packages\ipykernel_launcher.py:6: RuntimeWarning: invalid value encountered in double_scalars
```

# 7. Variability in the shapes of individual patches?¶

In [48]:

```
# regenerate centroids
ISI1_centroid_array_x = np.zeros((len(ISI1_list),len(mean_area_name_list)))
ISI1_centroid_array_y = np.zeros((len(ISI1_list),len(mean_area_name_list)))
ISI2_centroid_array_x = np.zeros((len(ISI1_list),len(mean_area_name_list)))
ISI2_centroid_array_y = np.zeros((len(ISI1_list),len(mean_area_name_list)))

for ii in range(len(ISI1_list)):
    for jj in range(len(mean_area_name_list)):
        ISI1_centroid_array_x[ii,jj] = scipy.ndimage.measurements.center_of_mass(ISI1_patch_mask_list[ii][jj])[0]
        ISI1_centroid_array_y[ii,jj] = scipy.ndimage.measurements.center_of_mass(ISI1_patch_mask_list[ii][jj])[1]
        ISI2_centroid_array_x[ii,jj] = scipy.ndimage.measurements.center_of_mass(ISI2_patch_mask_list[ii][jj])[0]
        ISI2_centroid_array_y[ii,jj] = scipy.ndimage.measurements.center_of_mass(ISI2_patch_mask_list[ii][jj])[1]

ISI1_centroid_array_x[ISI1_centroid_array_x == 0] = np.nan
ISI1_centroid_array_y[ISI1_centroid_array_y == 0] = np.nan
ISI2_centroid_array_x[ISI2_centroid_array_x == 0] = np.nan
ISI2_centroid_array_y[ISI2_centroid_array_y == 0] = np.nan

# x dimension is a-p axis with higher numbers corresonding to more posterior locations
# y dimension is m-l axis with higher numbers corresonding to more medial locations
```

In [49]:

```
def cv2_clipped_zoom(img, zoom_factor):
    """
    Center zoom in/out of the given image and returning an enlarged/shrinked view of 
    the image without changing dimensions
    Args:
        img : Image array
        zoom_factor : amount of zoom as a ratio (0 to Inf)
    """
    height, width = img.shape[:2] # It's also the final desired shape
    new_height, new_width = int(height * zoom_factor), int(width * zoom_factor)

    ### Crop only the part that will remain in the result (more efficient)
    # Centered bbox of the final desired size in resized (larger/smaller) image coordinates
    y1, x1 = max(0, new_height - height) // 2, max(0, new_width - width) // 2
    y2, x2 = y1 + height, x1 + width
    bbox = np.array([y1,x1,y2,x2])
    # Map back to original image coordinates
    bbox = (bbox / zoom_factor).astype(np.int)
    y1, x1, y2, x2 = bbox
    cropped_img = img[y1:y2, x1:x2]

    # Handle padding when downscaling
    resize_height, resize_width = min(new_height, height), min(new_width, width)
    pad_height1, pad_width1 = (height - resize_height) // 2, (width - resize_width) //2
    pad_height2, pad_width2 = (height - resize_height) - pad_height1, (width - resize_width) - pad_width1
    pad_spec = [(pad_height1, pad_height2), (pad_width1, pad_width2)] + [(0,0)] * (img.ndim - 2)

    result = cv2.resize(cropped_img, (resize_width, resize_height))
    result = np.pad(result, pad_spec, mode='constant')
    assert result.shape[0] == height and result.shape[1] == width
    return result
```

In [50]:

```
def find_optimal_rotation(input_array, reference_array):
    reference_array = reference_array.astype(np.bool)

    list_overlap = []
    for jj in range(360):
        temp = np.copy(input_array)
        seed = scipy.ndimage.measurements.center_of_mass(temp)
        rotation_matrix = cv2.getRotationMatrix2D((seed[1],seed[0]), jj, 1.0)
        temp = cv2.warpAffine(temp.astype(np.float), rotation_matrix, temp.shape).astype(np.bool)
        Jaccard_index = np.sum(np.bitwise_and(temp, reference_array)).astype(np.float) / \
                        np.sum(np.bitwise_or(temp, reference_array)).astype(np.float)
        
        list_overlap.append((Jaccard_index))
    optimal_rotation = np.argmax(list_overlap)
    rotation_matrix = cv2.getRotationMatrix2D((seed[1],seed[0]), optimal_rotation, 1.0)
    output_array = cv2.warpAffine(input_array.astype(np.float), rotation_matrix, input_array.shape)
    output_array[output_array > 0] = 1

    return optimal_rotation, output_array
```

In [51]:

```
# Test for biological variability of shapes of patches using the Jaccard Index (intersection / union).
# with scaling and rotation.

# Make a list of patches. Each is a binary mask.
patches_corrected = np.empty((60,11,2,600,600))
for jj in range(11):
    print jj, datetime.datetime.now()
    for ii in range(60):
        centered_patch = np.copy(mean_mask_list[jj])
        x_shift = 300 - scipy.ndimage.measurements.center_of_mass(centered_patch)[0]
        y_shift = 300 - scipy.ndimage.measurements.center_of_mass(centered_patch)[1]
        translation_matrix = np.float32([[1,0,y_shift],[0,1,x_shift]])
        centered_patch = cv2.warpAffine(centered_patch.astype(np.float), translation_matrix, centered_patch.shape)
        centered_patch = centered_patch.astype(np.bool)
        
        # ISI1
        patch = np.copy(ISI1_patch_mask_list[ii][jj].astype(float))
        if np.sum(patch) > 0:
            # center patch on 300,300
            x_shift = 300 - ISI1_centroid_array_x[ii,jj]
            y_shift = 300 - ISI1_centroid_array_y[ii,jj]
            translation_matrix = np.float32([[1,0,y_shift],[0,1,x_shift]])
            patch = cv2.warpAffine(patch, translation_matrix, patch.shape) 
            # scale patch
            scale_factor = np.sqrt(np.sum(centered_patch) / np.sum(patch)) # scale factor is the sqrt of the area ratio
            patch = cv2_clipped_zoom(patch, scale_factor)
            # rotate patch
            angle, patch = find_optimal_rotation(patch, centered_patch)
            # save patch
            patches_corrected[ii,jj,0,:,:] = patch
        else:
            patches_corrected[ii,jj,0,:,:] = 0

        # ISI2
        patch = np.copy(ISI2_patch_mask_list[ii][jj].astype(float))
        if np.sum(patch) > 0:
            # center patch on 300,300
            x_shift = 300 - ISI2_centroid_array_x[ii,jj]
            y_shift = 300 - ISI2_centroid_array_y[ii,jj]
            translation_matrix = np.float32([[1,0,y_shift],[0,1,x_shift]])
            patch = cv2.warpAffine(patch, translation_matrix, patch.shape) 
            # scale patch
            scale_factor = np.sqrt(np.sum(centered_patch) / np.sum(patch)) # scale factor is the sqrt of the area ratio
            patch = cv2_clipped_zoom(patch, scale_factor)
            # rotate patch
            angle, patch = find_optimal_rotation(patch, centered_patch)
            # save patch
            patches_corrected[ii,jj,1,:,:] = patch
        else:
            patches_corrected[ii,jj,1,:,:] = 0
patches_corrected = patches_corrected.astype(np.bool)

# test for biological variability of shapes of patches using the Jaccard Index (intersection / union)
for ii in range(len(mean_area_name_list)):
    # calculate the distribution of pairwise mouse-to-mouse differences
    diff_betw_mice = []
    for kk in range(len(ISI1_list)):
        for jj in range(kk + 1, len(ISI1_list)):
            if np.count_nonzero(patches_corrected[kk,ii,0]) > 0 and \
                                      np.count_nonzero(patches_corrected[jj,ii,0]) > 0:
                diff_betw_mice.append(np.sum(np.bitwise_and(patches_corrected[kk,ii,0], \
                                        patches_corrected[jj,ii,0])).astype(np.float) / \
                                      np.sum(np.bitwise_or(patches_corrected[kk,ii,0], \
                                         patches_corrected[jj,ii,0])).astype(np.float))
            if np.count_nonzero(patches_corrected[kk,ii,1]) > 0 and \
                                      np.count_nonzero(patches_corrected[jj,ii,1]) > 0:
                diff_betw_mice.append(np.sum(np.bitwise_and(patches_corrected[kk,ii,1], \
                                        patches_corrected[jj,ii,1])).astype(np.float) / \
                                      np.sum(np.bitwise_or(patches_corrected[kk,ii,1], \
                                        patches_corrected[jj,ii,1])).astype(np.float))
    diff_betw_mice = np.asarray(diff_betw_mice)

    # calculate distribution of ISI2-ISI1 differences
    diff_betw_measurements = []
    for kk in range(len(ISI1_list)):
        if np.count_nonzero(patches_corrected[kk,ii,0]) > 0 and \
                                      np.count_nonzero(patches_corrected[kk,ii,1]) > 0:
            diff_betw_measurements.append(\
                    np.sum(np.bitwise_and(patches_corrected[kk,ii,0], patches_corrected[kk,ii,1])).astype(np.float) / \
                    np.sum(np.bitwise_or(patches_corrected[kk,ii,0], patches_corrected[kk,ii,1])).astype(np.float))
    
    #stats
    _, p_val = scipy.stats.mannwhitneyu(diff_betw_mice, diff_betw_measurements)
    print '________________________'
    print ' '
    print mean_area_name_list[ii], 'p =', p_val
    if p_val < p_criterion / 11: # div by 11 for Bonferroni correction
        print 'p <', p_criterion / 11
    else:
        print 'no significant difference'
    print 'median mouse-to-mouse difference', np.nanmedian(diff_betw_mice)
    print 'median sessions-to-session difference', np.nanmedian(diff_betw_measurements)

    # plot
    fig = plt.figure(figsize=(20,15))
    
    ax1 = fig.add_subplot(221)
    hist_array = diff_betw_mice
    y, x, _ = ax1.hist(hist_array, bins=100, normed=True, alpha=0.5, range=(0,1))
    param = scipy.stats.lognorm.fit(hist_array, loc=0.7)
    x = np.linspace(0,1,100)
    pdf_fit = scipy.stats.lognorm.pdf(x, param[0], loc=param[1], scale=param[2])
    ax1.plot(x,pdf_fit,'b-', )
    
    hist_array = diff_betw_measurements
    y, x, _ = ax1.hist(hist_array, bins=100, normed=True, alpha=0.5, range=(0,1))
    param = scipy.stats.lognorm.fit(hist_array, loc=0.7)
    x = np.linspace(0,1,100)
    pdf_fit = scipy.stats.lognorm.pdf(x, param[0], loc=param[1], scale=param[2])
    ax1.plot(x,pdf_fit,'r-', )
    
    ax2 = fig.add_subplot(222)
    hist_array = diff_betw_mice
    y, x, _ = ax2.hist(hist_array, bins=100, normed=True, alpha=0.5)
    param = scipy.stats.lognorm.fit(hist_array, loc=0.7)
    x = np.linspace(0,1,100)
    pdf_fit = scipy.stats.lognorm.pdf(x, param[0], loc=param[1], scale=param[2])
    ax2.plot(x,pdf_fit,'b-', )
    
    hist_array = diff_betw_measurements
    y, x, _ = ax2.hist(hist_array, bins=100, normed=True, alpha=0.5)
    param = scipy.stats.lognorm.fit(hist_array, loc=0.7)
    x = np.linspace(0,1,100)
    pdf_fit = scipy.stats.lognorm.pdf(x, param[0], loc=param[1], scale=param[2])
    ax2.plot(x,pdf_fit,'r-', )
    ax2.set_xscale('log')

    plt.show()
```

```
0 2019-01-24 13:22:52.607000
1 2019-01-24 13:33:00.307000
2 2019-01-24 13:42:59.113000
3 2019-01-24 13:52:41.132000
4 2019-01-24 14:02:04.106000
5 2019-01-24 14:12:15.429000
6 2019-01-24 14:18:23.450000
7 2019-01-24 14:26:17.372000
8 2019-01-24 14:35:40.984000
9 2019-01-24 14:41:01.694000
10 2019-01-24 14:49:35.688000
________________________
 
V1 p = 0.0011886158652604742
p < 0.00454545454545
median mouse-to-mouse difference 0.8526911914779828
median sessions-to-session difference 0.8828944686440727
```

```
________________________
 
RL p = 0.46571273124109497
no significant difference
median mouse-to-mouse difference 0.5975992491787081
median sessions-to-session difference 0.5916382743950186
```

```
________________________
 
LM p = 0.0032995509068301657
p < 0.00454545454545
median mouse-to-mouse difference 0.7072609790822473
median sessions-to-session difference 0.7457722635510449
```

```
________________________
 
AM p = 0.00044154921536418206
p < 0.00454545454545
median mouse-to-mouse difference 0.7322046240669944
median sessions-to-session difference 0.7804714499554661
```

```
________________________
 
PM p = 0.0007285564121972877
p < 0.00454545454545
median mouse-to-mouse difference 0.6803305462205639
median sessions-to-session difference 0.7410815982194996
```

```
________________________
 
P p = 0.005241276022317555
no significant difference
median mouse-to-mouse difference 0.5321100917431193
median sessions-to-session difference 0.6156307841588138
```

```
________________________
 
RLL p = 0.11451279624734062
no significant difference
median mouse-to-mouse difference 0.7798329929933581
median sessions-to-session difference 0.7741770964830945
```

```
________________________
 
AL p = 1.625088877701347e-05
p < 0.00454545454545
median mouse-to-mouse difference 0.8046160877513712
median sessions-to-session difference 0.8479716790665818
```

```
________________________
 
LLA p = 0.2707894093274806
no significant difference
median mouse-to-mouse difference 0.7623165618448637
median sessions-to-session difference 0.7657409300273497
```

```
________________________
 
MMA p = 0.23349223862883756
no significant difference
median mouse-to-mouse difference 0.7608869356771873
median sessions-to-session difference 0.7564696507810273
```

```
________________________
 
MMP p = 0.24724220726808638
no significant difference
median mouse-to-mouse difference 0.7217200394441745
median sessions-to-session difference 0.7598268205843071
```

In [52]:

```
# no rotations
patches_corrected = np.empty((60,11,2,600,600))

for kk in range(11):
    centered_patch = np.copy(mean_mask_list[kk])
    x_shift = 300 - scipy.ndimage.measurements.center_of_mass(centered_patch)[0]
    y_shift = 300 - scipy.ndimage.measurements.center_of_mass(centered_patch)[1]
    translation_matrix = np.float32([[1,0,y_shift],[0,1,x_shift]])
    centered_patch = cv2.warpAffine(centered_patch.astype(np.float), translation_matrix, centered_patch.shape)
    centered_patch[centered_patch > 0] = 1

    for ii in range(60):
        # ISI1
        patch = np.copy(ISI1_patch_mask_list[ii][kk].astype(float))
        if np.nansum(patch > 0):
            # center patch on 300,300
            x_shift = 300 - scipy.ndimage.measurements.center_of_mass(patch)[0]
            y_shift = 300 - scipy.ndimage.measurements.center_of_mass(patch)[1]
            translation_matrix = np.float32([[1,0,y_shift],[0,1,x_shift]])
            patch = cv2.warpAffine(patch, translation_matrix, patch.shape)
            patch[patch > 0] = 1
            # scale patch
            scale_factor = np.sqrt(np.sum(centered_patch) / np.sum(patch)) # scale factor is the sqrt of the area ratio
            patch = cv2_clipped_zoom(patch, scale_factor)
            # save patch
            patches_corrected[ii,kk,0,:,:] = patch
        else:
            patches_corrected[ii,kk,0,:,:] = np.zeros((600,600))
        
        # ISI2
        patch = np.copy(ISI2_patch_mask_list[ii][kk].astype(float))
        if np.nansum(patch > 0):
            # center patch on 300,300
            x_shift = 300 - scipy.ndimage.measurements.center_of_mass(patch)[0]
            y_shift = 300 - scipy.ndimage.measurements.center_of_mass(patch)[1]
            translation_matrix = np.float32([[1,0,y_shift],[0,1,x_shift]])
            patch = cv2.warpAffine(patch, translation_matrix, patch.shape)
            patch[patch > 0] = 1
            # scale patch
            scale_factor = np.sqrt(np.sum(centered_patch) / np.sum(patch)) # scale factor is the sqrt of the area ratio
            patch = cv2_clipped_zoom(patch, scale_factor)
            # save patch
            patches_corrected[ii,kk,1,:,:] = patch
        else:
            patches_corrected[ii,kk,1,:,:] = np.zeros((600,600))

patches_corrected = patches_corrected.astype(np.bool)

diff_betw_mice = np.zeros((11,600,600))
diff_betw_measurements = np.zeros((11,600,600))

# test for biological variability of shapes of patches using the Jaccard Index (intersection / union)
for ii in range(11):
    centered_patch = np.copy(mean_mask_list[ii])
    x_shift = 300 - scipy.ndimage.measurements.center_of_mass(centered_patch)[0]
    y_shift = 300 - scipy.ndimage.measurements.center_of_mass(centered_patch)[1]
    translation_matrix = np.float32([[1,0,y_shift],[0,1,x_shift]])
    centered_patch = cv2.warpAffine(centered_patch.astype(np.float), translation_matrix, centered_patch.shape)
    centered_patch = centered_patch - scipy.ndimage.morphology.binary_erosion(centered_patch, iterations=1)
    centered_patch[centered_patch == 0] = np.nan
    centered_patch[np.isfinite(centered_patch)] = mean_field_sign_list[ii]
    
    count = 0
    count2 = 0
    # calculate the distribution of pairwise mouse-to-mouse differences
    for kk in range(len(ISI1_list)):
        for jj in range(kk + 1, len(ISI1_list)):
            if np.count_nonzero(patches_corrected[kk,ii,0]) > 0 and \
                                      np.count_nonzero(patches_corrected[jj,ii,0]) > 0:
                diff_betw_mice[ii,:,:] = diff_betw_mice[ii,:,:] + np.bitwise_or(patches_corrected[kk,ii,0], \
                                        patches_corrected[jj,ii,0]).astype(np.float) - \
                                      np.bitwise_and(patches_corrected[kk,ii,0], \
                                        patches_corrected[jj,ii,0]).astype(np.float)
                count = count + 1
            if np.count_nonzero(patches_corrected[kk,ii,1]) > 0 and \
                                      np.count_nonzero(patches_corrected[jj,ii,1]) > 0:
                diff_betw_mice[ii,:,:] = diff_betw_mice[ii,:,:] + np.bitwise_or(patches_corrected[kk,ii,1], \
                                        patches_corrected[jj,ii,1]).astype(np.float) - \
                                      np.bitwise_and(patches_corrected[kk,ii,1], \
                                        patches_corrected[jj,ii,1]).astype(np.float)
                count = count + 1     

    # calculate distribution of ISI2-ISI1 differences
        if np.count_nonzero(patches_corrected[kk,ii,0]) > 0 and \
                                      np.count_nonzero(patches_corrected[kk,ii,1]) > 0:
            diff_betw_measurements[ii,:,:] = diff_betw_measurements[ii,:,:] + \
                    np.bitwise_or(patches_corrected[kk,ii,0], patches_corrected[kk,ii,1]).astype(np.float) - \
                    np.bitwise_and(patches_corrected[kk,ii,0], patches_corrected[kk,ii,1]).astype(np.float)
            count2 = count2 + 1
    
    diff_betw_mice[ii,:,:] = diff_betw_mice[ii,:,:] / count
    diff_betw_measurements[ii,:,:] = diff_betw_measurements[ii,:,:] / count2
    

    print '________________________'
    print ' '
    print mean_area_name_list[ii]

    # plot
    fig = plt.figure(figsize=(20,20))
    ax1 = fig.add_subplot(221)
    ax2 = fig.add_subplot(222)
    
    ax1.imshow(diff_betw_mice[ii,150:450,150:450], cmap='Greys', interpolation='nearest', zorder=0)
    ax1.imshow(centered_patch[150:450,150:450], clim=(-1,1), cmap='bwr', interpolation='nearest', alpha=1, zorder=1)
    ax1.tick_params(
        axis='x',          # changes apply to the x-axis
        which='both',      # both major and minor ticks are affected
        bottom='off',      # ticks along the bottom edge are off
        labelbottom='off') # labels along the bottom edge are off
    ax1.tick_params(
        axis='y',          # changes apply to the y-axis
        which='both',      # both major and minor ticks are affected
        left='off',        # ticks along the bottom edge are off
        labelleft='off')   # labels along the bottom edge are off
    
    ax2.imshow(diff_betw_measurements[ii,150:450,150:450], cmap='Greys', interpolation='nearest', zorder=0)
    ax2.imshow(centered_patch[150:450,150:450], clim=(-1,1), cmap='bwr', interpolation='nearest', alpha=1, zorder=1)
    ax2.tick_params(
        axis='x',          # changes apply to the x-axis
        which='both',      # both major and minor ticks are affected
        bottom='off',      # ticks along the bottom edge are off
        labelbottom='off') # labels along the bottom edge are off
    ax2.tick_params(
        axis='y',          # changes apply to the y-axis
        which='both',      # both major and minor ticks are affected
        left='off',        # ticks along the bottom edge are off
        labelleft='off')   # labels along the bottom edge are off

    plt.show()
```

```
________________________
 
V1
```

```
________________________
 
RL
```

```
________________________
 
LM
```

```
________________________
 
AM
```

```
________________________
 
PM
```

```
________________________
 
P
```

```
________________________
 
RLL
```

```
________________________
 
AL
```

```
________________________
 
LLA
```

```
________________________
 
MMA
```

```
________________________
 
MMP
```
